# CVD Environmental Health Disparities Tool: systematic evidence mapping of psychosocial stressors, environmental exposures, and cardiovascular diseases to inform disparities research and action

**DOI:** 10.1186/s12940-026-01269-9

**Published:** 2026-02-26

**Authors:** Ruth M. Lunn, Katherine R. Helmick, Grace Cooney, Meredith Clemons, Melissa Polansky, Samantha J. Snow, Wren Tracy, Darlene Dixon

**Affiliations:** 1 National Institute of Environmental Health Sciences, P.O. Box 12233, MS K2-14, Research Triangle Park, RTP, Durham, NC 27709 USA; 2 ICF, Reston, VA USA

**Keywords:** Cardiovascular disease, Environmental health disparities, Psychosocial stressors, Environmental exposures, Systematic evidence maps

## Abstract

**Background:**

Globally, cardiovascular disease (CVD) is the leading cause of death and disproportionately affects the poor and certain races and ethnic groups.

**Objectives:**

Identify and map current knowledge on contributors, i.e., psychosocial stressors (PSS) and environmental exposures, to these disparities. PSS may work though the same mechanistic pathways and could also increase the susceptibility of an individual to environmental exposures.

**Methods:**

We conducted systematic evidence mapping constructed from searches performed in PubMed. Study data was extracted for environmental exposures, PSS, CVD-related outcomes, disproportionately affected populations (DAPs), and other characteristics. Finally, we selected four exemplars two environmental exposures (noise, heat/cold), one PSS (discrimination), and one CVD (allostatic load) with varying databases to conduct a more in-depth review and illustrate the utility of the tool.

**Results:**

We created the CVD Environmental Health Disparities Tool using data from over 8,000 relevant reviews and primary studies identified from over 53,000 unique references. The publicly available tool consists of six interactive systematic evidence maps (SEMs); three are specific for DAPs and three for all populations. Collectively, these SEMs include 35 environmental exposures, 13 PSS, and > 30 CVD outcomes in 9 populations. Each SEM allows users to visualize and filter the evidence by the extracted data, evaluate patterns, and identify research gaps and identify recommendations to meet those gaps. We found few studies on minority sexual orientation populations, household products, and green spaces. To illustrate the utility of the tool, for each exemplar, we identified recommendations for primary studies, e.g., the need to evaluate the interactions between PSS and environmental exposures (all exemplars), conduct studies in DAPs (e.g., noise, heat/cold for racial and ethnic groups), and determine which exposure-outcome pairs warranted a systemic review (e.g., discrimination and several CVDs) or intervention (e.g., allostatic load could be used to evaluate intervention efficacy).

**Conclusion:**

Our comprehensive systematic evidence mapping and tool is unique because it provides a comprehensive characterization of studies of environmental exposures and PSS across all CVD outcomes. It is a resource that can be leveraged to inform effective research leading to action and provide knowledge to affected communities and community-based organizations.

**Supplementary Information:**

The online version contains supplementary material available at 10.1186/s12940-026-01269-9.

## Background

 Cardiovascular disease (CVD) is the leading cause of death in individuals living in the United States and worldwide [1]. Significant racial, ethnic, and geographical health disparities in CVD mortality, disease, and age of onset are observed in disproportionately affected populations (DAPs), which often consist of Black/African American, Asian, Hispanic, and Indigenous people [2–10]. The causes of health disparities in CVD are multifactorial and can be due to psychosocial stressors (PSS) attributable to differences in risk factors (e.g., genetics/epigenetics, personal lifestyle behaviors, socioeconomic status), community factors (e.g., social structure), and social or societal factors (e.g., access to health care, institutional policies) [11–18]. Environmental exposures (e.g., air pollution, pesticides, metals) can also contribute to CVD risk and these exposures are often more prevalent in communities of color that are more likely to be impacted by chronic PSS, which are implicated in increased susceptibility to environmental contaminants [19–22].

 The role of environmental exposures and PSS in explaining health disparities is a notable research gap [23]. Modifying factors such as stress, age, diet, and genetics are important elements for integrating environmental exposures in the CVD framework, especially in DAPs. Health disparities could also be attributed to higher exposure levels, or the interaction between environmental exposures and PSS, or both. Knowledge of the state of the science is needed, e.g., published studies of environmental exposures, PSS, and CVD in DAPs, to inform the implementation of effective CVD health disparities research leading to impactful interventions and quality health. An approach for obtaining this knowledge is systematic evidence maps (SEMs). These are novel tools that allow users to explore large databases, e.g., researchers can quickly determine the number of published studies for a specific interest, such as given exposure and CVD exposure-outcome pair, and identify data gaps and inform research initiatives.

### Objective and goals

Our primary objective is to develop an interactive tool to identify and map the environmental and psychosocial contributors to CVD disparities to facilitate knowledge mining, effective epidemiological research, and interventions. This tool consists of several interactive SEMs (e.g., databases characterizing the relevant studies), which can also be a resource for members of DAPs and community-based organizations to gain knowledge. For example, the SEMs can serve as a source of information that scientific communities can use to facilitate participatory research partnerships between scientists and community-based organizations or inform policies. These interactions can assist the advancement of research on CVD and environmental health disparities and help promote environmental health that is equitable with just solutions for all. A secondary aim is to map environmental exposure studies of CVD in the general population to identify data gaps and provide context for the DAP studies.

Our publicly available CVD Environmental Health Disparities Tool (consisting of multiple SEMs) highlights the findings for specific bodies of studies that may be of interest to DAPs and other socially marginalized groups and identifies research gaps in this field.

## Methods

### Identifying the literature: problem formulation, strategy, and searches

In consultation with a library specialist, we developed a broad literature search strategy for PubMed to capture literature relevant to the effects of environmental exposures and PSS on CVD in both general populations and DAPs. We used PubMed only as the purpose was to identify health studies and gaps and were not conducting a systematic review to reach formal health hazard conclusions. We completed a series of separate literature searches using a combination of environmental exposure, PSS, CVD outcome, epidemiological, and DAP terms to target relevant primary articles and reviews. The original searches we conducted in 2020 retrieved over 100,000 citations. To reduce these large search results to a manageable number of references to screen, we adjusted our main search strategy to conduct six different searches focused on the study objective (to identify environmental exposure and PSS studies in DAPs) and restricted the search dates for each search depending on the exposure (i.e., environmental, PSS), population (i.e., DAP, general population) and article type (i.e., reviews, primary) as we were more interested in targeting DAP-focused literature. These six searches included: (1) environmental exposure and CVD primary studies limited to 2017, (2) environmental exposure and CVD reviews limited to 2015, (3) environmental exposure and CVD studies and reviews in DAPs limited to 2010, (4) PSS and CVD primary studies in DAPs limited to 2017, (5) PSS and CVD reviews in DAPs limited to 2015, and (6) PSS and environmental exposures and CVD studies and reviews in DAPs limited to 2010. Revised updated searches were conducted in 2022 and 2023 for focused topics of interest (e.g., discrimination, heat/cold, noise pollution), and in 2023 and 2025 for all topics. A summary of the literature search parameters (Table S1) and the complete literature search strings (Tables S2–S7) are available in the Supplemental Materials. PubMed search results were compiled in an EndNote library and duplicate studies were removed.

We developed an additional literature search to capture primary and review articles on allostatic load (AL) in both humans and animal models; this search was not date limited due to the small number of results. AL is a measure of a composite of cardiovascular-, neuroendocrine-, metabolic- and immune-system biomarkers that reflect multisystemic physiological dysregulation caused by cumulative stress, including that from daily life and major chronic events [24].

### Creating the CVD Environmental Health Disparities Tool: study selection, characterization, and systematic evidence mapping

#### Study selection

To identify relevant primary epidemiology studies and reviews, we conducted title-abstract screening in SWIFT-Active Screener (Sciome) [25] using the population, exposure, comparator, and outcome (PECO) criteria listed in Table 1 and the additional inclusion and exclusion criteria described in Table S8. The inclusion/exclusion criteria provided additional guidance for screeners on inclusion (e.g., include meta-analyses and non-English studies) and exclusion (e.g., do not include intervention studies, treatment studies, case series, case reports). Two independent screeners reviewed each study until SWIFT-Active Screener’s machine learning algorithm estimated at least 95% of relevant literature was included in each screening group [26]. Direct conflicts between screeners were tracked in SWIFT-Active Screener and resolved via third-party discussion among senior members of the team.

**Table 1:** PECO criteria

**P**opulation	**E**xposure	**C**omparators	**O**utcome
Humans: all populations^*a*^	*Environmental exposures*: air pollution, allergens, consumer exposures, environmental pollutants, metals, occupational exposures, physical agents, shift work, sleep quality, smoking (effect modifier), heat, noise, and others	No or low exposure to the environmental exposure	CVD biomarkers (including allostatic load^*b*^), pre-CVD (organ or subclinical such as hypertension, atherosclerosis, heart rate variability), specific CVD (e.g., DAD, PAD, combined CVD (e.g., incidence, mortality), clinical events (e.g., cardiac arrest, heart failure, stroke, MI)
	*Psychosocial stressors*: discrimination, education, food insecurity, geography, green space, SES, allostatic load,^*b,c*^ and others	No or low exposure to the psychosocial stressor	
Humans: DAPs	*Environmental exposures*: air pollution, allergens, consumer exposures, environmental pollutants, metals, occupational exposures, physical agents, shift work, sleep quality, smoking (effect modifier), heat, noise, and others	No or low exposure to the environmental exposure	
	*Psychosocial stressors*: discrimination, education, food insecurity, geography, green space, SES, allostatic load,^*b,c*^ and others	No or low exposure to the psychosocial stressor	

*CAD* coronary artery disease, *CVD* cardiovascular disease, *DAP* disproportionately affected population, *MI* myocardial infarction, *PAD* pulmonary artery disease, *SES* socioeconomic status

^*a*^For studies on allostatic load, animal populations were also included

^*b*^In all populations and DAP studies, allostatic load was included as a possible exposure or a possible outcome

^*c*^For studies that looked at allostatic load as a stressor in DAPs, a CVD outcome was not required for inclusion

After completion of title-abstract screening, studies deemed relevant or unclear at the title-abstract level were retrieved for full-text review. We performed full-text review and tagging in the Health Assessment Workspace Collaborative (HAWC) web-based content management system [27, 28]. One screener reviewed and tagged each study, and a second expert reviewer reviewed 30% of the studies for quality control. The PECO criteria outlined in Table 1 were used to determine relevancy at the full-text level along with additional detailed inclusion and exclusion criteria specified in Table S9. The inclusion/exclusion criteria provided additional guidance for screeners on inclusion (e.g., include meta-analyses and non-English studies) and exclusion (e.g., do not include serum measurements, like cholesterol, or post-CVD outcomes like post-stroke treatment or rehabilitation). Detailed categorization or tagging criteria conducted at the full-text level can be found in Table S10.

#### Study characterization

During the tagging process, screeners reviewed and applied tags to each relevant study in HAWC to denote the subcategories (i.e., specific environmental exposures, PSS, cardiovascular outcomes, and populations) and other study aspects (i.e., study design, geographical location, and women-specific studies) that each study covered. We organized CVD outcomes by disease progression, ranging from biomarkers (e.g., events that can occur in cells or tissues) to pre-CVD (e.g., organ, subclinical), CVD (e.g., incidence, mortality), and clinical events (e.g., myocardial infarction, cardiac arrest).

#### Systematic evidence mapping

The full-text tagging results were summarized in SEMs to explore the data by the different relevant topic groups using Tableau (Seattle, WA) [29]. The results were exported from HAWC and processed using Microsoft Excel and Tableau into six SEMs following the HAWC organization scheme (see Table 2 and Results and Discussion).

**Table 2:** Six Systematic Evidence Maps in the CVD Environmental Health Disparity Tool

		SEM Columns		
		All Populations		
SEM Rows	Environmental Exposures	Psychosocial Stressors	Cardiovascular Disease	DAP
Environmental Exposures	-	-	-	DAP x ENV
Psychosocial Stressors	ENV x PSS	-	-	DAP x PSS
Cardiovascular Disease	ENV x CVD	PSS x CVD	-	DAP x CVD

All six SEMs in the CVD Environmental Health Disparities Tool are available on Tableau [30]

Filters enable exploration of CVD x ENV x All Populations (or DAP) in all SEMs. All SEMs also have filters below the tables for ENV, PSS, CVD, CVD outcome details, All Populations or DAP, study type, geographical location, women only, and study list. Table topics are repeated in the table as a 2nd way to filter (pull down menu) but partial lists are not visible without using the menu

*CVD* cardiovascular disease, *DAP* disproportionately affected population, *ENV* environmental exposures, *PSS* psychosocial stressors, *SEM* systematic evidence map

#### Exemplars or highlighted research

To illustrate how users can use the tool to identify research gaps and make recommendations, we examined reviews and primary studies for topics of interest for each of the main components of our SEM: noise pollution and heat or cold for environmental exposures, discrimination for PSS, and allostatic load for CVD. The exemplars were selected because they represented exposures and PSS that may be of special interest to our DAP groups and play a potential role in health disparities and potentially had data gaps that differ for DAP groups. AL was selected as it may be part of the causal pathways between environmental exposures and CVD outcomes. Primary studies and reviews for each topic were identified using the SEMs' filters. For selected primary studies, we extracted the findings and more detailed study data, such as the study population, exposure, CVD outcome, or PSS (for example, see Supplemental Table 11 for information on discrimination studies). Due to the large number of studies on heat (or cold) and noise pollution, our review focused on primary studies in DAP or reporting on both exposure and PSS. We did not conduct a systematic review that evaluated biases and integrated the evidence across studies to reach a CVD hazard conclusion. However, for relevant exposure (or PSS) CVD outcome pairs, we did report whether the evidence (based on the number of studies) was positive or null for exposure and conclusions from any systematic reviews. Finally, we made research recommendations for primary studies, systematic reviews, and intervention or policy.

## Results and discussion

### Overview

#### CVD Environmental Health Disparities Tool

##### Study selection

**Fig. 1:**
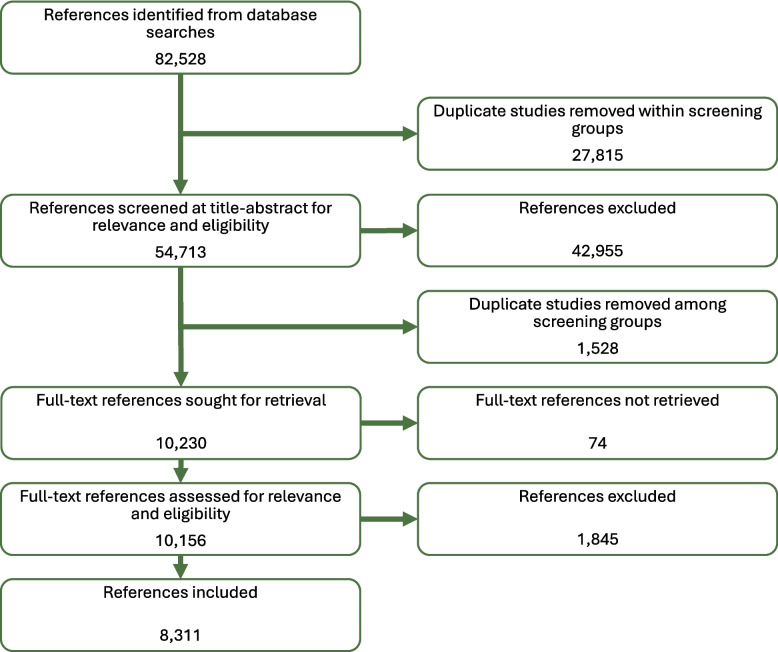
PRISMA Diagram. Depicts the study selection flow for the systematic evidence mapping of psychosocial stressors, environmental exposures, and cardiovascular diseases. Studies that did not meet the PECO criteria (Table 1) were excluded; additional inclusion and exclusion criteria are listed in the Supplemental Material (Table S8 and Table S9). There were two rounds of deduplication of references due to how the screening was conducted: references were deduplicated within each of their respective screening groups prior to title/abstract screening, and references were deduplicated amongst the different screening groups after title/abstract screening was completed

Fig. 1 depicts the Preferred Reporting Items for Systematic Reviews and Meta-Analyses (PRISMA) findings for the literature searches. After removing duplicate studies within each search group, 54,713 citations (across all searches) were identified for title-abstract screening. Title-abstract screening resulted in 42,955 excluded citations and full-text review excluded another 1,845 citations. Ultimately, 8,311 references were included in our review.

##### Tool organization

The CVD Environmental Health Disparities Tool includes data from 6,904 primary studies and 1,409 reviews (note that several studies conducted both primary studies and reviews which is why these numbers do not add up to 8,311). The tool landing page provides information on the project purpose, and links to the methods, dashboard help, definitions, and visualization of the six interactive SEMs (Table 2). Interactive versions of the SEMs and instructions for using the CVD Environmental Health Disparities Tool can be viewed online in Tableau [30]. Three SEMs contain data from all populations and three SEMs have data specific for DAPs (see Table 2) and multiple SEMs were created to focus on our main topics, environment exposure, PSS, CVD, and population and facilitate the users’ experience for answering research questions.

**Fig. 2:**
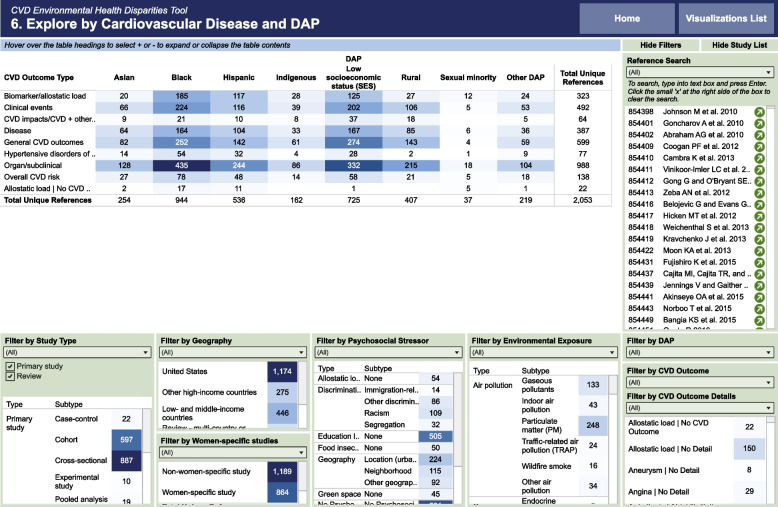
Cardiovascular Diseases (CVD) Environmental Health Disparities Tool. This screenshot is of one of six SEMs (CVD X DAP SEM) [31] in our CVD Environmental Health Disparities Tool, which aims to explore potential causes (environmental exposures and PSS) of CVD that may disproportionately affect some populations. The heatmap columns are different DAPs; the rows are the main CVD categories organized by disease progression that can be expanded to display individual outcomes (e.g., hypotension under subclinical, coronary artery disease under diseases, stroke under clinical events). In addition to the heatmap, the user can filter the results by different study characteristics: study type, geographic regions, women-specific studies, environmental exposures, PSS, and CVD outcomes. The study list updates to reflect filtering applied. Other SEMs in the tool show heatmaps comparing different categories and the results in all publications. The CVD Environmental Health Disparities Tool [30] provides background and help information and allows results to be downloaded. This screenshot is filtered on primary studies

Each SEM presents the data in a main table that is based on the title tables with filters of all study elements below or to the right of the table. For example, the CVD and DAP table (Fig. 2) has CVD outcomes as rows and DAPs as columns. CVD rows can be expanded to show the many different CVD outcomes in the broader group (e.g., the category clinical effects include angina, cardiac rest, heart failure, myocardial infarction, and stroke). As mentioned in the methods, CVD outcomes are grouped by disease progression: biomarkers, pre-CVD (organ or subclinical), CVD (specific CVD such as CAD and combined CVD such as mortality), and clinical events (myocardial infarction). The category “CVD risk” includes a mixture of biomarkers and pre-diseases (such as hypertension).

SEM filters include study elements, including study type (e.g., review, cross-sectional), geographical location (e.g., United States, low- and middle-income countries), population (e.g., DAPs and women), environmental exposures (grouped by types), PSS (grouped by types), and detailed CVD outcomes and endpoints. Some publications reported both a review and primary study. We also included studies on CVD impact (e.g., disability-adjusted life years and years of life lost). Users can filter on preclinical endpoints and the potentially related disease(s) (e.g., heart rate variability and arrhythmia) and on specific details of the cardiovascular disease or endpoint (e.g., different types of arrhythmias, atrial fibrillation, or ventricular electrical abnormalities). Interactive versions of the SEMs and instructions for using the CVD Environmental Health Disparities Tool can be viewed online in Tableau [30].

We use the tool to answer our broad research objective of identifying research gaps and evaluating patterns for environmental exposures, PSS, and CVD outcomes in different populations. For each topic, we then highlight research on the exemplar and make recommendations regarding research gaps.

#### All studies

Our review identified 6,904 relevant primary epidemiological studies of CVD, including 4,078 on environmental exposures (“environmental-exposures-only studies”), 1,950 on PSS (“PSS-only studies”), and 876 on PSS and environmental exposures. Few studies in the last category reported interactions between PSS and environmental exposures, however. We also included studies on allostatic load that were not tagged for a CVD outcome. As part of our detailed data extraction for discrimination (see the Discrimination exemplar in the PSS section below), we identified several cohorts that may be informative for evaluating CVD health disparities because they stratified or focused on DAPs (highlighted in Table 3).

**Table 3:** Examples of Cohorts Reporting on Disproportionately Affected Populations

Focus	Cohort	Additional Information
General Health	ELSA-Brazil (Brazilian Longitudinal Study of Adult Health)	https://pubmed.ncbi.nlm.nih.gov/22234482/ [32]
	SWAN (Study of Women's Health Across the Nation)	https://www.swanstudy.org/ [33]
Cardiovascular Diseases	CARDIA (Coronary Artery Risk Development in Young Adults)	https://www.nhlbi.nih.gov/science/coronary-artery-risk-development-young-adults-study-cardia [34]
	Jackson Heart Study	https://www.jacksonheartstudy.org/ [35]
	MESA (Multi-Ethnic Study of Atherosclerosis)	https://www.mesa-nhlbi.org/ [36]
	HCHS/SOL (Hispanic Community Health Study/Study of Latinos Sociocultural Ancillary Study)	https://sites.cscc.unc.edu/hchs/node/400 [37]
Psychosocial Stressors	Add Health (National Longitudinal Study of Adolescent to Adult Health)	https://www.icpsr.umich.edu/web/ICPSR/series/1006 [38]
Health Disparities	EHDIC (Exploring Health Disparities in Integrated Communities Study)	https://pure.johnshopkins.edu/en/publications/exploring-health-disparities-in-integrated-communities-overview-o-4 [39]

#### DAP studies

Because CVD rates vary by both income and race, we selected seven populations that may be disproportionately affected to environmental exposures or PSS based on race/ethnicity (i.e., Black, Asian, Hispanic, and Indigenous people), income, sexual orientation or identity (i.e., sexual minority), and place (i.e., people living in rural areas). Examples of other DAPs include other racial ethnic groups (e.g., Arab-American, Middle Eastern) and non-White (not specific) people.

We found 1,786 primary studies reporting on CVD and environmental exposure or PSS in DAPs (Fig. 2, also includes studies of allostatic load without a CVD outcome). Most of these studies were conducted among Black people (*N *= 813), Hispanic people (*N* = 478), and people with lower socioeconomic status (SES) (*N* = 603). Indigenous people (*N* = 120) were the least studied racial and ethnic group, and sexual minorities were the least studied DAP (*N* = 27).

More than 850 CVD primary studies reported on environmental exposures, over 1,250 primary studies reported on PSS, and 344 of these primary studies reported on both these exposures. We noted several differences in study elements between the environmental exposure and PSS studies. Studies of cardiovascular clinical diseases and events (61% vs. 44%), low- and middle-income country (LMIC) locations (31% vs. 16%), and women (58% vs. 37%) were more frequent in environmental exposure-only studies than in PSS-only studies.

The distribution of studies across the various study characteristics for the different DAPs followed similar patterns, but there were some differences. Most studies (> 90%) of Black and Hispanic people were of people living in the United States. Compared with these two groups, a higher percentage of studies of Asian (22%) and Indigenous people (40%) were conducted outside the United States, mainly in other high-income countries (HIC). Studies of rural populations were more common in LMICs (70%), whereas those examining low-income populations were reported more in all HIC (73%). Almost half the primary studies (across DAPs) reported risks for women only, with a somewhat lower percentage reporting risks for Indigenous people (32%).

#### Highlighted research

As mentioned in the methods, we selected four exemplars, two for environmental exposures (noise, heat/cold), one PSS (discrimination), and one CVD outcome (allostatic load), for which we conducted a more in-depth review and made recommendations for the need for primary studies, systematic reviews, and or interventions. The recommendations for each exemplar are discussed in the major topic section (i.e., Environmental Exposures, PSS, CVD outcomes) and summarized across exemplars in the Discussion.

### Environmental exposure studies

#### All studies

**Fig. 3:**
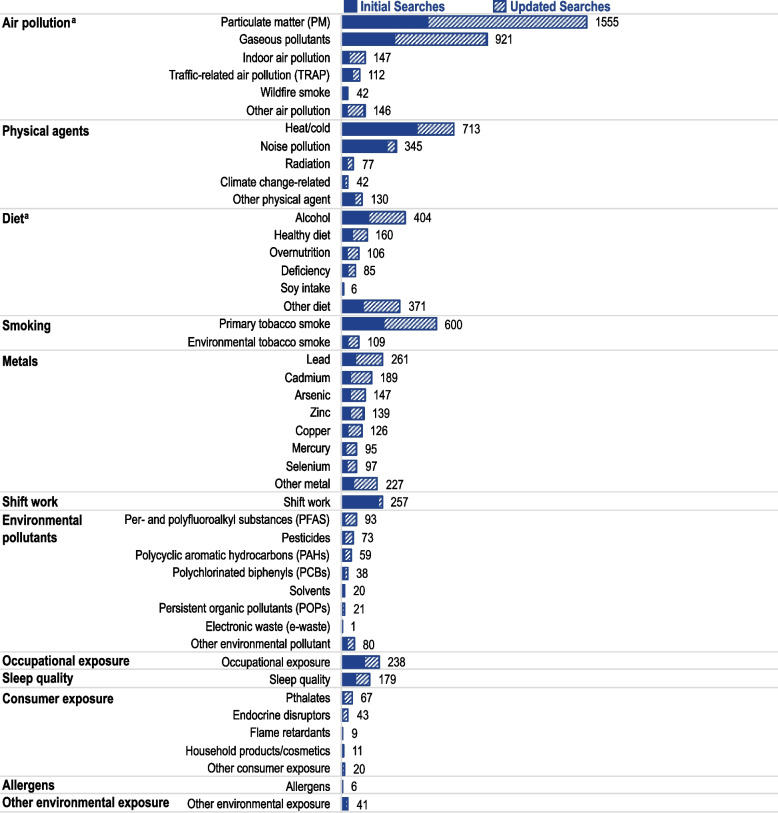
Environmental Exposures Reported for All Populations in Primary Studies Identified During Initial Searches and Updated Searches. Figure 3 shows the number of primary epidemiological studies by environmental exposures for all studies included in the review. The solid color represents the original searches and the hatch bars represent the updated searches. The figure was created using data from the Environmental Exposures x Cardiovascular Diseases in All Populations SEM [40] in the CVD Environmental Health Disparities Tool. ^a^The updated searches underestimate air pollution and diet and enrich for the special topics of interest (i.e., sleep, noise, heat, and cold)

Our review includes almost 5,000 studies of more than 35 different types of environmental exposures categorized into 12 groups (see Fig. 3 and Environmental Exposures x Cardiovascular Disease in All Populations SEM [40]. We considered diet (which included alcohol consumption), smoking, and sleep quality to fall under personal behaviors; the other categories were related to exposures from the external environment, from the workplace, or from using consumer products. The bulk of the research focused on well-established CVD risk factors, including outdoor air pollution, personal behaviors, and certain metals, as well as more recently identified risk factors, such as heat exposure related to climate variability or shifts. Our SEMs may underestimate the number of air pollution and diet studies as the updated literature searches contained general but not specific search terms for air pollution given that these are established risk factors. Fewer studies are available on consumer products and environmental pollutants. Within consumer exposures, the bulk of the studies are for endocrine disruptors and phthalates; growing evidence suggests that prenatal exposure to phthalates may affect cardiovascular risk or hypertension in a gender-specific manner that may be influenced by puberty [41, 42]. The relationship between phthalates and hypertension may be modified by genetic or behavior [43]. Per- and polyfluoroalkyl substances were the most reported environmental pollutants with approximately half the studies examining hypertension.

#### DAP studies

**Fig. 4:**
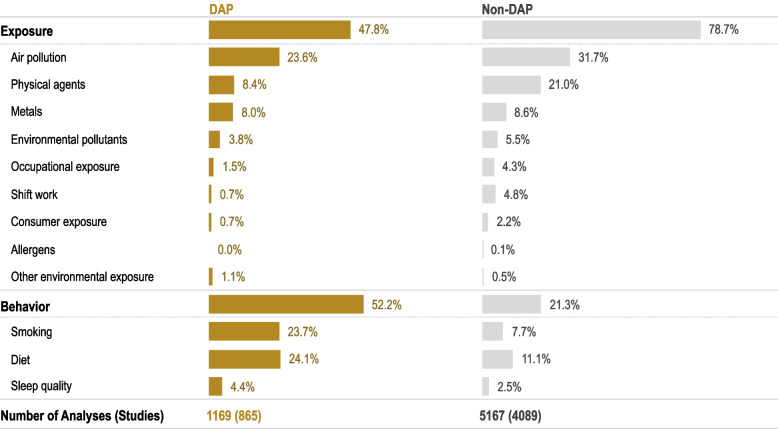
Environmental Exposures in Primary Studies of Disproportionally Affected Populations (DAPs) and General Populations. Because many studies reported on more than one exposure, this figure shows the percentage of environmental exposure analyses (e.g., number of studies that reported analyses for a specific environmental exposure divided by total number of environmental exposure analyses) for both DAP and non-DAP (e.g., those studies that did not report results specific for DAP). Although DAP and non-DAP populations had different search dates, the denominator is for the population group. The figure was created using data (using the population filters to obtain number of studies) from the Environmental Exposures x Cardiovascular Diseases in All Populations SEM [40] in the CVD Environmental Health Disparities Tool

Only 18% of all environmental exposure primary studies reported findings for DAP and CVD outcomes despite the longer literature search inclusion date for DAP studies. In general, the distribution of these studies parallels non-DAP-specific studies (i.e., most studies were on established risk factors, such as personal behaviors and air pollution). However, our SEMs suggest there may be a bias or a general population/DAP gap in evaluating environmental exposures: 79% of non-DAP studies reported on environmental exposure compared with 48% in DAPs (Fig. 4). Because there were differences in search dates for DAP and non-DAP, we conducted a sensitivity analysis using the original search restricted to similar dates (2017 to 2020) and found comparable results (e.g., similar trend as the full data).

**Fig. 5:**
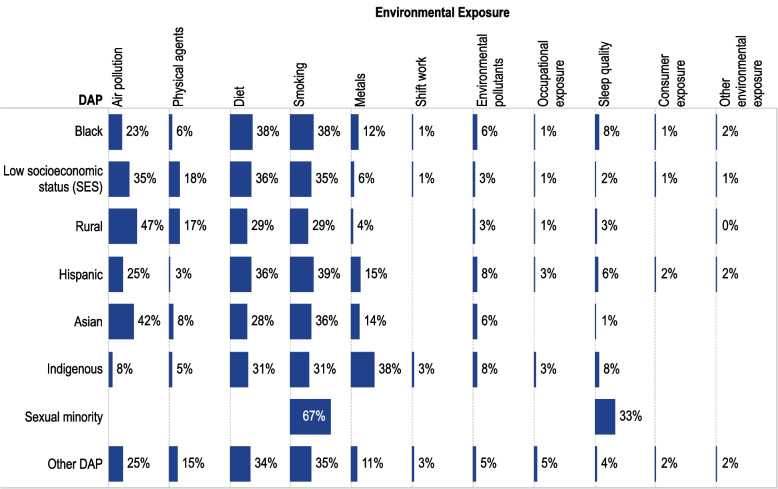
Distribution of Primary Studies of Environmental Exposures Across Disproportionally Affected Populations (DAPs). Figure 5 shows the percentage of primary studies that reported on the association of an environmental exposure (columns) within a specific DAP (rows) (e.g., number of studies of specific environmental exposure divided by total studies of a specific DAP). Data do not add up to 100% because some studies report on several environmental exposures. The figure was created using data from the Environmental Exposures x DAP SEM [44] in the CVD Environmental Health Disparities Tool. ^a^The updated searches underestimate air pollution and diet and enrich for the special topics of interest (i.e., sleep, noise, heat, and cold)

There were also differences in the distribution of studies across DAPs (Fig. 5 and Environmental Exposures x DAP SEM [44] in the CVD Environmental Health Disparity Tool). Air pollution was a more commonly examined exposure in studies of Asian people and rural communities (accounting for almost half the studies of each DAP) than in other groups (23% to 35% of all environmental exposure per DAP), less common in studies of Indigenous people (8%), with no studies looking at the effect of air pollution in sexual minorities. In contrast, studies of metals were more frequent among Indigenous people (38% of all environmental exposures for this group) than with other groups (4% to 15% of all environmental exposures per group and no studies examining sexual minority populations). Climate-related studies (i.e., heat and cold, within the physical agents’ category) were more common in lower SES and rural groups (12% to 14%, respectively, of environmental exposures) than in the ethnic and racial DAPs (~ 3–4 of environmental exposures and no studies examining sexual minority populations). As mentioned above, air pollution and diet studies may be underestimated in the updated search because of the removal of air pollution and diet-specific search terms in the literature search updates conducted in 2023 and 2025; however, sensitivity analysis (using number of studies) restricted to the original search showed similar patterns, and thus the differences in search strategy by dates did not impact our evaluation of these trends.

#### Highlighted research

We chose two exemplar environmental exposures that may be higher in DAPs to demonstrate how the tool can be used to identify research gaps and make recommendations (Table 5). Our review did not assess study quality. For these reviews, we used all populations and DAP SEMs, to identify the relevant studies.

#### Noise pollution

##### Review

We identified 533 studies that examined noise exposure, including 347 primary studies and 187 reviews. The literature reviews and primary studies in our review support the role of noise pollution (from airplanes, roads, railroads, and occupational sources) as a risk factor for various CVD outcomes and identify a research need for studies to evaluate health disparities from noise exposure.

Of the 347 primary studies identified, only nine examined the effect of noise exposure on CVD among DAPs. Four studies examined associations between noise and CVD in specific DAP populations and five studies examined noise-CVD associations stratified by specific DAPs. Across the nine studies, race/ethnicity, SES, and geographic location were considered. Overall, the findings are difficult to interpret given the small number of heterogeneous studies examining different populations, age groups, and CVD effects.

Four studies examined the impact of race or ethnicity on the association between noise and CVD outcomes, either by studying specific racial or ethnic groups [45, 46] or stratifying analyses by race/ethnicity [47, 48]. Although only a small number of studies were identified, positive findings were reported across these studies of various populations (e.g., Māori, Black, South Asian, other), life stages (e.g., adults, children), exposures (e.g., occupational noise, residential noise, airplane noise), and CVD outcomes (e.g., ischemic heart disease, systolic blood pressure, CVD-related hospitalization), suggesting that the association between noise and CVD effects may be of concern for certain racial or ethnic groups.

Five studies evaluated the impact of SES, with two studies examining noise exposure and CVD effects in low SES populations [46, 49] and three studies stratifying analyses by SES [50–52]. Although there were few studies, the majority reported positive associations for low SES groups. The two studies in low SES populations both examined blood pressure, with positive associations in a study of children [46] and inverse associations in a study of low-income housing adults [49]. In studies stratifying by SES, associations for noise exposures (occupational, daytime, or nighttime) and CVD outcomes were significantly positive (atrial fibrillation and CVD events) [51, 52] or null (hypertension) [50] among low SES groups. There was no evidence of effect modification by SES.

Two studies stratified analyses of nighttime noise exposure and CVD outcomes (stroke, hypertension) by geographic location [50, 52], with increased risk of stroke among rural populations compared to urban [52] and no significant difference for adult hypertension in rural and urban populations [50]. Additional studies are needed to assess the impact of rurality on the association between noise and CVD.

Overall, the data are difficult to interpret given the heterogeneous database on the association between noise exposure and CVD among DAPs. More evidence is needed. Using a geospatial sound model to estimate regional noise pollution, Casey et al. [53] found higher estimated nighttime and daytime noise levels in census tracts with higher proportions of non-Whites and lower SES residents in the United States. Proposed mechanisms for noise-related CVD effects include increases in stress hormones and vascular oxidative stress, which lead to endothelial dysfunction and hypertension [54]. PSS could reasonably augment noise-induced CVD risk via these mechanisms, which could also lead to health inequities.

Of the 94 studies examining noise and PSS, only one study investigated the combined effect of noise and PSS. Klompmaker et al. [55] found no association between joint exposure to both noise (≤ 52 decibels (dB) L_den_ [daily active noise level]) and green space (300 m Normalized Difference Vegetation Index; OR: 1.03, 95% CI: 1.00–1.07) on hypertension. However, this study also reports that traffic noise does not affect risk of hypertension, which contradicts the preponderance of evidence identified in this review that supports the association between exposure to traffic noise and increased risk of hypertension. As such, more research is needed to understand how PSS may influence the association between noise and CVD risk.

The predominance of noise pollution research was conducted outside the United States. Globally, the World Health Organization strongly recommends reducing noise levels produced by road traffic to an average exposure of below 53 dB L_den_. The U.S. Environmental Protection Agency (EPA) [56] is responsible for researching noise-related health effects and distributing the findings to the public, responding to inquiries, and evaluating the effectiveness of existing regulations for protecting public health and welfare. However, since 1981, noise control has been delegated to state and local governments. Systematic assessments of local and state regulations may be needed to determine whether noise regulations are based on nuisance or on health effects and whether there are any geographical inequalities (Table 5).

#### Heat and cold

##### Review

Our review identified 713 primary studies and 170 reviews that evaluated the relationship between hot and/or cold temperatures and CVD outcomes. Overall, growing evidence in the reviews and studies indicates an association between ambient heat exposure and increased CVD risk in general populations. In a recent review and meta-analysis, Liu et al. [57] found that a 1 °C increase in temperature was associated with increased CVD-related mortality. The overall risk of CVD-related mortality increased by 2.1% for every 1 °C increase in temperature, with the highest risk for stroke and coronary heart diseases, and heatwaves were significantly associated with an 11.7% increased risk of CVD-related mortality. Liu et al. [57] also found that 1 °C increase in temperature was associated with significant increases in morbidity due to arrythmias, cardiac arrest, and coronary heart disease. The authors identified certain populations as having an elevated risk of CVD-related morbidity and mortality caused by heat, including women, people 65 years and older, individuals living in tropical climates, and those living in LMICs. The review did not specifically look at heat exposure and CVD risk in DAPs. In another recent meta-analysis that stratified results by countrywide SES, Wen et al. [58] found that hot ambient temperature was significantly associated with a 10% increase of stroke morbidity and 9% increase in stroke mortality, whereas cold ambient temperature was associated with a 33% increase in the risk of stroke morbidity and 18% increase in stroke mortality. The authors found stroke morbidity and mortality from heat was higher in HICs than in LMICs. Conversely, the effect of cold ambient temperatures on stroke morbidity and mortality was shown to be lower in HICs and higher in LMICs.

Our analysis focused on heat/cold studies specifically in DAPs, either in studies with a DAP as the study population or studies with the temperature-CVD results stratified by DAP. We identified 67 primary studies that assessed the effects of heat/cold on CVD in DAPs. The majority of studies (80%) were ecologic or another study design (e.g., time-series, case-crossover), 10% were cross-sectional, and 10% were cohort. The study investigators evaluated the heat and cold exposures with a variety of metrics, including heat waves/extreme heat events [59–73], cold spells/extreme cold events [60, 68, 70, 73–76], temperature variability [77–79], thermal indeices [80–82], heat stress index levels [83], increased/decreased mean daily apparent or ambient temperature [67, 84–101], daily 3-h maximum apparent temperature [102, 103], same-day maximum/minimum temperature [104], mean monthly temperature [105], increased/decreased mean annual ambient temperature [106, 107], increased/decreased hourly ambient temperature [108], short-term mean ambient temperature [109], warmest days of summer or coldest days of winter [81, 110], mean winter/summer environmental temperatures [111, 112], increase/decrease above/below threshold temperatures [113], heat effects or cold effects [87, 114–119], diurnal temperature range [120, 121], perceived temperature [122], and cold indoor temperatures [123].

For the purposes of our high-level analysis, we classified these studies into three overarching categories: heat effects, cold effects, and temperature variability. However, the diversity in temperature metrics adds additional nuance to the exposure analysis of these studies—like effects of short-term vs. long-term temperature effects, seasonal trends, and scope of temperature change (e.g., extreme heat vs. slight increases in heat)—which are not fully covered in our analysis. Most studies on DAPs focused on heat-CVD effects (79%), whereas 52% looked at cold-CVD effects and four studies (6%) assessed temperature variability-CVD effects. The majority of studies focused on the temperature-CVD relationships in low SES DAPs (51%) and rural DAPs (48%), while fewer studies assessed racial and ethnic DAPs like Black (15%), Hispanic (6%), Asian (5%), Indigenous (3%), and other unspecified non-White populations and ethnic minorities (16%). Geographically, 19% occurred in the United States, 39% of studies occurred in other HIC, and 42% occurred in LMIC.

Our summary of primary studies in DAPs suggests heat/cold is associated with an increased risk of several adverse CVD outcomes in DAPs. The relationship between heat and CVD was assessed in 53 studies. Among outcomes evaluated in at least 10 studies, the evidence points to positive associations between heat and increased myocardial infarctions (79% of studies) and all CVD (e.g., CVD-related mortality, incidence, and hospitalization; 63% of studies) in DAPs (Table 4). Among outcomes discussed in fewer DAP studies (5–10; see Table 4), heat appears to be related to increased risk of coronary artery disease (88% of studies), cerebrovascular disease (71% of studies), and stroke (57% of studies). We examined the effects of cold on CVD outcomes in DAPs in 35 primary studies, however, only two CVD outcomes were assessed in more than 5 studies. Similar to heat, the studies suggest cold is associated with an increased risk of myocardial infarctions (positive associations in 80% of studies; *N* = 10) [81, 89, 99, 109, 115, 118, 119, 124] and all CVD (positive associations found in 76% of studies; *N* = 17) [74–76, 80, 81, 84, 86, 107, 113, 116, 122–124]. Temperature variability was only assessed in four studies in DAPs and these found mixed [78, 79] or positive [77, 121] associations with CVD outcomes.

**Table 4:** Summary of Studies Examining the Relationship between Heat and CVD Outcomes in DAPs

Number of Studies	CVD Outcome (# of Studies)	Summary of Findings on Heat-CVD Effects in DAPs
> 10 studies	CVD-related incidence, hospitalization, or mortality (32)	↑ Positive: 63% positive [59, 61, 63, 66, 69, 72, 81, 83, 84, 86, 92, 98, 100, 101, 107, 110, 113, 114, 120, 124]
		↓ Negative: 16% negative [80, 94, 102, 103, 116]
		↓↑ Mixed findings: 13% mixed findings [62, 70, 88, 90]
		− Null: 9% null [65, 95, 105]
5–10 studies	Coronary artery disease (8)	↑ Positive: 88% of studies [68, 81, 88, 94, 100, 115, 124]
		↓ Negative: 1 study [106]
		↓↑ Mixed findings: 1 study [62]
5–10 studies	Cerebrovascular disease (7)	↑ Positive: 71% of studies [81, 100, 102, 103, 124]
		↓ Negative: 1 study [88]
		− Null: 1 study [95]
> 10 studies	Myocardial infarction (14)	↑ Positive: 79% of studies [60, 64, 65, 71, 81, 93, 100, 109, 115, 118, 124]
		↓ Negative: 1 study [119]
		− Null: 2 studies [95, 99]
5–10 studies	Stroke (7)	↑ Positive: 57% of studies [87, 94, 120, 125]
		↓ Negative: 29% of studies [88, 106]
		− Null: 1 study [95]

*CVD* cardiovascular disease, *DAPs* disproportionately affected populations

##### Recommendations

Epidemiological evidence has established a link between climate-related exposures—air pollution and heat—and increased CVD mortality and morbidity. It is well established that extreme weather change is an environmental fairness issue warranting the need for policy changes and intervention. Intervention and policy may benefit from (1) knowledge about whether PSS, including access to green space, urban versus. rural location, and SES, modify the effects of heat/cold exposure on CVD; (2) additional research on the effects of heat/cold in DAPs, especially racial and ethnic minorities; (3) studies of precursors to CVD (e.g., changes in ejection function); and (4) research on interventions to reduce the effects of heat and cold exposure (Table 5).

**Table 5:** Research Recommendations for Environmental Exposures, PSS, and CVD Exemplars

Topic	Current Research Status	Recommendations: Primary Studies	Recommendations: Systematic Reviews	Intervention and Policy
Noise Pollution 9 DAPs	Risk factors for several CVD outcomes Few studies in DAP	Studies in DAP PSS x environmental exposure interactions	None recommended at this time	Evaluate the effectiveness of noise regulations to protect CVD health
Heat and Cold 67 DAPs; 21 in racial and ethnic groups	Growing evidence related to several increased CVD outcomes in DAP (rural and low SES)	Studies in racial and ethnic DAPs PSS x environmental exposure interactions	DAP for selected outcomes	Intervention studies in DAP
Discrimination 193 all, 149 DAPs^a^	Adequate database Suggested associations: AL, biomarkers, combined CVD Possibly hypertension and stroke	Cohort studies, other CVD outcomes, sexual minorities PSS x environmental exposure interactions Pathway analysis	AL, hypertension, precursors, combined CVD and stroke Meta-analysis and attributed risk: Hypertension, combined CVD outcomes	Interventions, such as community support, anti-biased policies, and actions
Allostatic Load 292 all, 128 DAPs	↑Risk for CVD and in Black people Associated with low SES, adverse childhood experience, discrimination Few environmental studies May measure “weathering"	Environmental studies, including PSS interactions Animal studies	None recommended at this time	AL can be used to evaluate the efficacy of interventions

*AL* allostatic load, *CVD* cardiovascular disease, *DAP* disproportionately affected population, *PSS* psychosocial stressors; *SES* socioeconomic status

^a^Does not include studies on immigration that are not measuring discrimination per se

### Psychosocial stressors

#### All studies

 Our review identified more than 2,800 primary studies reporting on eight PSS categories and found a wealth of studies evaluating SES, education, and place (i.e., geographical location such as neighborhood, urbanicity). An additional category captured other PSS that did not fit within the eight categories (e.g., perceived stress, maternal stress, childhood adversity, non-SES specific vulnerability indices). Data is from the Psychosocial Stressors x Cardiovascular Disease in All Populations SEM [126] in the CVD Environmental Health Disparity Tool. An area of growing interest is food insecurity. Landsbergis et al. (2024) [127] noted that inadequate attention has been given to work-related stress role in CVD; this is especially important as workers are also exposed to higher levels of chemicals or mixtures. Although many other PSS categories (e.g., green spaces, work-related stress, neighborhood) were reported in more than 100 studies, there were research gaps for certain CVD outcomes as most of the studies evaluated biomarkers, pre-CVD, or combined CVD outcomes, and fewer studies evaluated specific CVD outcomes or clinical events. AL is a biomarker of cumulative stress and represents PSS (and potential risk factors for CVD) or presents as an outcome (see CVD Outcomes section, below). Although our searches for PSS contained search terms for DAPs, we included studies that were not specific for DAPs that were picked up by general terms. Thus, the PSS in the general population is an underestimate of the total studies.

#### DAP studies

**Fig. 6:**
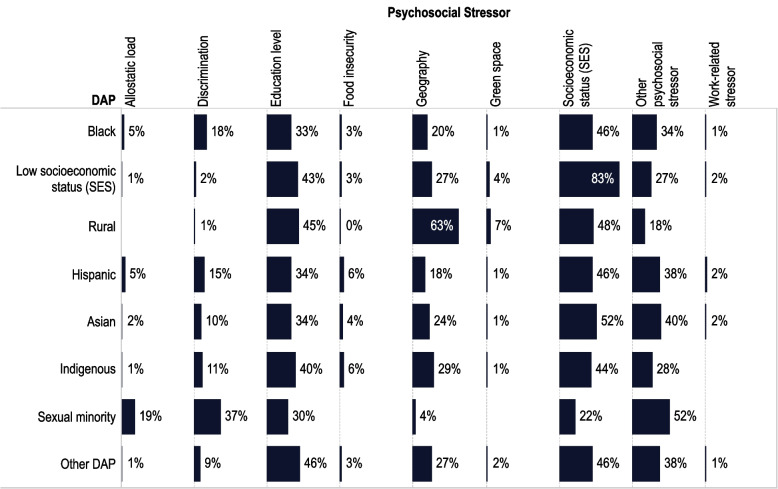
Distribution of Primary Studies of Psychosocial Stressors Across Disproportionally Affected Populations (DAP). Figure 6 shows the percentage of primary studies that reported on the association of a psychosocial stressor (columns) within a specific DAP (rows) (e.g., number of studies of specific PSS divided by total studies of a specific DAP). Data do not add up to 100% because some studies report on several PSS. The figure was created using data from the Psychosocial Stressors x DAP SEM [126] in the CVD Environmental Health Disparities Tool

 Patterns of PSS examined in DAP were similar to those of all studies; however, some PSS, such as work-related stress may be less examined in DAP (*N* = 20 studies). Discrimination was the most evaluated specific PSS among sexual minorities (37%) and was reported more in studies focused on Black (18%) and Hispanic people (15%) than on Asian people (10%) or Indigenous to 11%) and infrequently examined in people having lower SES (2%) or living in rural communities (1%) (Fig. 6 and the Psychosocial Stressor x DAP SEM [128] in the CVD Environmental Health Disparity Tool). The smaller number of studies for some DAPs make this comparison somewhat unreliable.

#### Highlighted research: discrimination

##### Review

We identified 193 primary studies (not including studies of immigrants) that evaluated the relationship between discrimination and CVD outcomes. Table S11 provides a summary of the evidence across studies organized by CVD outcome and characterized by several features (i.e., type of study design, positive or null findings, and relevant information such as type of discrimination, surrogates, effect modifiers and any environmental exposures). Most of the evidence is from U.S. studies (90%) and in DAPs (77%), with over half the studies conducted among Black people; few studies reported on sexual minorities. Many studies (56%) were cross-sectional analyses, often using cohort data collected at baseline; 30% of the studies were cohort studies. Study investigators measured perceived discrimination, including its frequency (e.g., every day) and duration (e.g., lifetime), using validated or standard tools such as the Experiences of Discrimination (EOD) scale [129], Schedule of Racist Events (SRE) [130] and the Everyday Discrimination Scale (EDS) [131]. In studies that focused on the reason for discrimination, race/ethnicity was most common; however, some assessed body weight [132–134], gender [133, 135], and sexual orientation [17, 136–138]. Institutional racial bias and segregation were more challenging to assess and sometimes were measured using proxies such as excessive use of police force, e.g., Freedman et al. 2022 [139], negative Twitter tweets, e.g., Huang et al. 2020 [140], historical slavery, e.g., Kramer et al. 2017 [141], redlining, Al-Kindi et al. 2023, Jadow et al. 2023, Motairek et al. 2022, Mujahid et al. 2021, Wing et al. 2022 [142–146], skin color, e.g., Wassink et al. 2017 [147], living in an area with hate crime [148], and other surrogates of racial bias, e.g., Basile-Ibrahim et al. 2021, Stanhope et al. 2023, Siegel et al. 2023 [149–151]. A few experimental or randomized studies exposed participants to racial bias (e.g., written vignettes of racial biased behaviors) [152–154] or sexual orientation bias (e.g., interviewers with anti-gay attitudes) [137] (Table S11).

Our summary of primary studies suggests that discrimination is associated with an increased risk of several adverse cardiovascular outcomes (Table S11). Among outcomes evaluated in at least 15 studies, the evidence points to associations (based on a large majority of studies, i.e., > 80%, reporting positive findings) with AL and CVD-related biomarkers, Associations may be less consistent (71 to 77%) for hypertension, CVD risk factors, combined CVD mortality or incidence, and stroke. Although examined in fewer studies (five to nine), discrimination was associated with atherosclerosis-related or cardiac-related early events and heart failure in 80% of the studies. Other outcomes had few studies, or findings across studies were mixed. In general, more studies looking at racial or ethnic discrimination indicated a positive association than did studies that assessed other types of discrimination; however, there were few studies that examined the same type of discrimination. Some studies evaluated indirect (e.g., discrimination as an intermediate) rather than direct effects or effect modifiers with other PSS. Although some studies also evaluated environmental exposure and CVD outcomes, only four evaluated whether discrimination modified the risk from environmental exposure—sleep [155] and air pollution [156–158].

Our review is not a comprehensive systematic review that reaches hazard conclusions (e.g., whether discrimination is causally associated with a given CVD outcome). Although we categorized the studies by various features, we did not assess study quality or perform a formal stratified analysis of the strength of the evidence by study design, population, discrimination type (e.g., cumulative, every day) or source of biased action (e.g., race/ethnicity, weight, gender, sexual orientation). We also examined the reviews in our SEM to determine whether adequate systematic reviews were evaluated for the CVD outcomes compared with the primary studies. Panza et al. [159] found that 86% of studies published up to 2018 found a positive association between discrimination and CVD-related outcomes (primarily cardiovascular biomarkers but not including AL) and hypertension among socially stigmatized groups. The authors also assessed other indicators of cardiovascular health; most of the studies were focused on risk factors (e.g., weight, diabetes, history of CVD) and only a few studies included actual outcomes. Although they conducted a study quality evaluation, the authors used a risk-of-bias tool that did not follow best practice, did not assess how bias affects the evidence, and did not reach hazard conclusions. Advantages of the Panza et al. review were stratification for different sources of discrimination (e.g., race, weight, sexual orientation). For AL, a systematic review of 11 studies concluded that discrimination was associated with this outcome [160]; however, this report did not include many primary studies in our SEM (outside its literature search range) or adequately evaluate the complex indirect patterns related to sex and race suggested by some primary studies or effect modifiers (Table S11). Other systematic reviews for hypertension were older, e.g., Dolezsar et al. (2014) and Couto et al. (2012) [161, 162]. Reviews for other CVD outcomes were for specific populations, e.g., older African Americans [163], young African American women [164], or sexual minority [165]; specific types of discrimination, e.g., redlining [166], or experimental studies of exposure to racial or non-racial stressors [167]. Agbonlahor et al. (2023) [168] conducted a systematic review that reported on the number of studies (including percent positive) examining racial or ethnic discrimination and cardiometabolic diseases conducting in United States but did not conduct evaluate study informativeness or have SEM. The Agbonlahor review overlaps partially with our exemplar, however, the type of discrimination and geographical location is more limited, and the CVD outcomes differ, and it does not include an interactive SEM.

##### Recommendations

We recommend new systematic reviews with rigorous study quality assessment and evidence integration methods, and meta-analyses if appropriate. We also recommend reaching hazard conclusions and characterizing uncertainty for several CVD outcomes with at least seven studies, including AL, hypertension, other CVD precursors (i.e., atherosclerosis and cardiac related), combined CVD morbidity, mortality, and stroke (see Table 5). These reviews should consider the impact of bias on the study findings and explore contributors of heterogeneity, such as bias, DAP category (e.g., race, gender, sexual orientation), type of discrimination (e.g., every day, childhood, cumulative), and effect modifiers or pathways (e.g., coping, other PSS). The evidence from our systematic evidence mapping suggests that discrimination experiences increase AL; however, a systematic review may shed light on pathways and effect modification by coping, other PSS, race, and gender. The John Henryism hypothesis [169] postulates that high coping efforts, especially in populations with inadequate resources to overcome stress, may be associated with a higher risk of adverse outcomes, such as hypertension and other CVD-related outcomes. A couple of studies in our review have evaluated interactions of discrimination and coping on AL in Black women. AL, as an early biomarker of effect, could be used to evaluate intervention strategies to prevent CVD. For hypertension, combined CVD, and possibly stroke, quantitative effect estimates could be used to calculate the attributable risk for discrimination. Systematic review for stroke and other outcomes may help to identify sources of heterogeneity in these studies. Primary studies, ideally cohort or prospective studies, are needed to evaluate other CVD outcomes, environmental exposures and discrimination interactions, and specific DAPs, such as sexual minorities (see Table 5).

### CVD outcomes

**Fig. 7:**
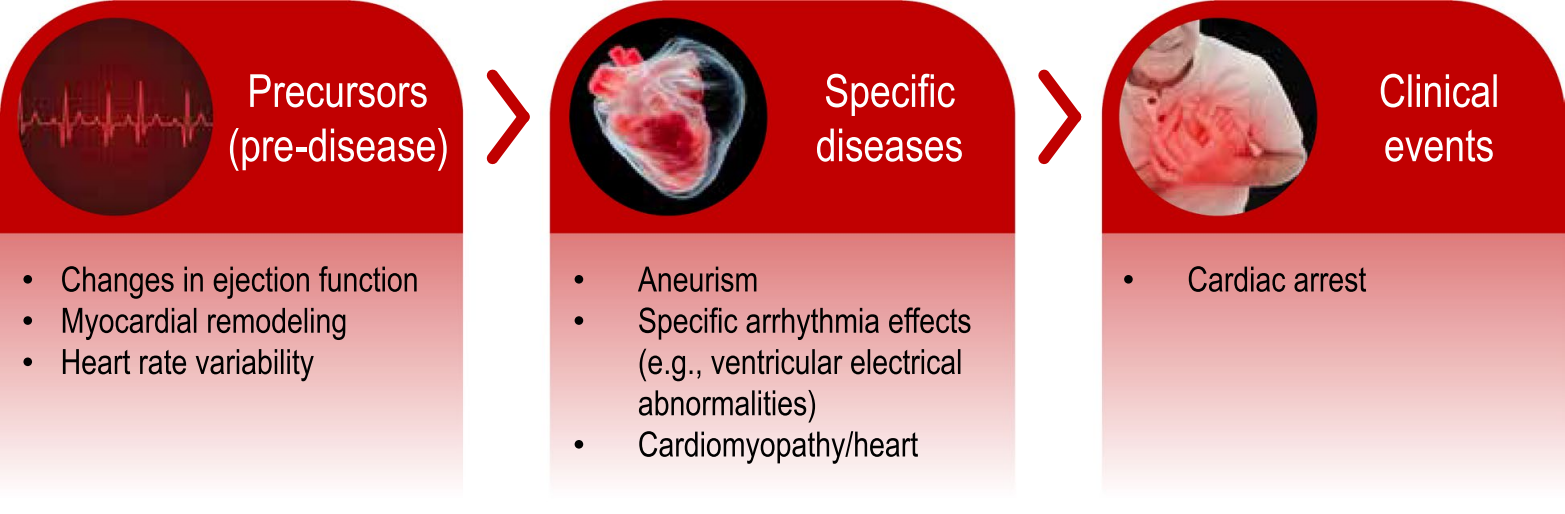
Cardiovascular Disease (CVD) Research Gaps. Figure 7 provides research gaps for specific CVD research gaps organized by disease progression. Gaps were identified from the number of studies reported for each CVD outcome in the CVD Health Disparities Tool, Environmental Exposure x CVD in All Populations SEM [40] and Psychosocial Stressors x CVD in All Populations SEM [126]

Our research shows that the distribution of CVD outcomes in DAPs and all populations are similar and may reflect in part the prevalence of specific CVDs and outcome data availability (see Environmental Exposures x CVD in All Populations SEM [40], the Psychosocial Stressors x CVD in All Populations SEM [126], and the CVD x DAP SEM [31] in the CVD Environmental Health Disparity Tool). About two-thirds of the studies reported on hypertension (36%) or combined CVD incidence or mortality (30%). CAD was the most reported disease (15% of all studies) and stroke was the most common clinical outcome (16%) of all studies. Slightly more than 10% of the studies reported on biomarkers, which included three main categories: (1) AL, (2) oxidative stress and inflammation, and (3) other biomarkers. More PSS studies (44%) reported on findings on hypertension compared with environmental exposure studies (37%), whereas more environmental exposure studies reported on specific CVDs, such as cerebrovascular disease, CAD, stroke, and myocardial infarction than did PSS studies. Figure 7 identifies research gaps for CVD for the different progression states except for biomarkers, for which we did not conduct a deeper dive.

#### Highlighted research: allostatic load (AL)

As mentioned in the Methods, AL is a measure of a composite of multi-system biomarkers of physiological dysregulation caused by cumulative stress [24]. High AL in children is associated with poor health outcomes [170] and several cohort studies in our review evaluating AL as a stressor found higher AL was associated with an increased risk of CVD [171–173] and specific CVD outcomes (e.g., CAD, heart failure) [174, 175]. We chose this outcome as an exemplar because it may represent intermediate pathways leading to health disparities [176] and could be used to measure the efficiency of interventions (see Table 5). We identified 292 studies on AL (published since 2010), most of which evaluated its association with PSS, especially SES and education. These studies or reviews indicate that AL is higher among Black people and is associated with lower SES (including lower childhood SES), neighborhood SES, adverse childhood experience (ACE) [177–179], and discrimination (see Highlighted Research: Discrimination Studies above). Several studies suggest that the relationship between PSS (such as childhood or neighborhood SES) can be mediated or moderated by health risk behaviors, education, social support, and coping strategies [24, 179] and suggest starting interventions early.

About 15% of all the identified AL studies evaluated the relationship between environmental exposures and AL, and most of them examined personal or health risk behaviors. These studies suggest that AL may be linked to personal behaviors (e.g., alcohol, smoking, diet) [170, 180], sleep deprivation [181–187], built environment (such as less green space, noise) [170], artificial light at night [188], and pollution or chemicals, including metals [189], per- and polyfluoroalkyl substances [190, 191], occupational (military exposure) and air pollution [115, 192, 193].

The relationship between exposures, PSS, and AL may depend on interactions between exposures and PSS. For example, Tomfohr et al. [155] reported a significant association between race and AL: experiencing discrimination led to anger, which resulted in worsening subjective sleep quality (personal behavior) and, ultimately, increased AL. Further research is needed to replicate these findings and better understand the impact of interactions between environmental exposures and PSS on AL (see Table 5).

Disparities across many types of health conditions are thought to be partially explained by chronic exposure to economic and social disadvantage that results in accelerated decline in health outcomes, which is known as the weathering hypothesis [194]. AL is thought to contribute to weathering due to chronic stress over a lifetime that results in adverse physiological outcomes. Weathering can result in the speeding up of aging and an earlier onset of decline in physical health conditions and is observed among disadvantaged people more than in advantaged people of similar age (i.e., weathering pattern) [195–199]. Results from a systematic review showed that weathering-related age patterns were observed for most health outcomes [194]; however, there were varied results depending on which comparison group was studied. Of the studies comparing health outcomes by age in U.S. Black people compared with White people, all but four studies (on preterm birth [200], cholesterol level [201], cardiovascular disease [202], and functional limitations [203]) observed evidence of weathering. Studies in which AL is a focused primary health outcome reported that weathering occurs when race or ethnicity [204–208] or SES [206, 208] are used as measures of disadvantage. Also, in additional studies that considered AL as a primary exposure [209–211], evidence of weathering was demonstrated by higher AL being associated with increased overall mortality rates and lower gestational age [211].

Our literature search for AL was not limited to humans and found several studies in nonhuman primates. These nonhuman primate studies support the findings from human studies that demonstrated stress is associated with AL and that AL is associated with increased risk for all causes of mortality and morbidity but that there was an inverse association with CVD [212]. In free-ranging rhesus monkeys, the authors concluded that stress associated with low dominance rank in a nonhuman primate society may be similar to having low SES in human societies [213]. Higher AL was associated with lemurs housed in smaller average group sizes and kept indoors and in those animals that experienced fewer changes in group composition [214].

## Strengths and limitations

 Our systematic evidence mapping and resulting CVD Environmental Health Disparities Tool have several strengths. They are comprehensive in scope and include studies on a broad range of environmental exposures and PSS, both of which can be causes of environmental health disparities. The Tool consists of six interactive SEMs and includes multiple filters that allow users to explore the data in many ways. Our review is inclusive for many types of cardiovascular outcomes, which are organized by progression from biomarker to event (e.g., biomarker, organ/subclinical endpoints, clinical diseases, clinical syndromes, and all CVD outcomes). Tags capture a streamlined data extraction as we reviewed the full text of 6,904 primary studies. For four topics of interest (i.e., noise, heat/cold, discrimination, AL), we extracted more details to summarize findings and identify research gaps. We recognize that there are many other exemplars that we encourage users to explore. For example, our review identified 238 studies on occupational exposures, of which only 18 studies were in DAP. We also identified 123 studies on work-stress, including 26 in DAP. A review (in our SEM) noted that many occupational exposures (such as metals, PAHs, mixtures) and psychosocial stressors related to work are associated with several CVDs [215]. Evaluating potential interactions between work-stress, environmental exposures, and DAP may advance cardiovascular health science in general and reduce potential health-related disparities.

 Our search strategy with different inclusion dates has both strengths and limitations. As previously mentioned, given the large database (from the initial search) and the need to review every study, we restricted the dates of our search strategy. We biased the strategy (i.e., longer search dates) to focus on our objectives of identifying environmental exposure and PSS studies in DAPs. We also aimed to identify environmental exposures that are risk factors for CVD in general; we therefore supplemented our shortest search strategy with reviews to obtain the desired information. Our 2023 and 2025 updated literature searches helped to minimize the differences in the total years searched for specific topics. Given the large number of studies, our updated literature searches focused on emerging topics and did not use the exposure-specific terms for well-established CVD risk factors (diet and air pollution); however, some air pollution studies continued to be captured due to our general environmental search terms. These differences in the multiple literature searches potentially present challenges in comparing across SEMs, and to a lesser extent comparing some environmental exposures in the general population SEM. However, our sensitivity analyses using the original search or restricting the studies to similar dates found patterns similar to the complete data. We contend these limitations are compensated for by the advantage of having more effectively used our resources to update a large body of literature to focus on our primary objective of identifying health-related disparities research gaps. 

 Systematic reviews are informative for reaching health hazard conclusions and informing evidence-based policy. Our exemplars illustrate several advantages of including reviews in our report: (1) identifying literature outside the literature search dates (e.g., discrimination and hypertension reviews found primary studies that evaluated effects of exposure to bias due to race or ethnicity, performing tasks, and hypertension not found in our SEM); (2) focusing the reviews of large databases to identify research gaps (e.g., by first examining reviews, we found 187 noise reviews and 170 heat/cold reviews were linked to CVD outcomes), allowing us to focus on a more limited database [e.g., studies in DAPs] and evaluating PSS and environmental exposure interactions); and (3) facilitating research recommendations on whether systematic reviews and possible meta-analyses are needed (e.g., the absence of available systemic reviews for discrimination and several cardiovascular outcomes). In contrast, the reviews for examining patterns in individual SEMs were not helpful in part because of less precise or “over tagging” of broad narrative reviews that were due to many different exposures and CVD outcomes and thus were not used. For future SEMs, we recommend limiting review to systematic or narrative-focused reviews that provide detailed study information. 

 We also recognize other limitations. As we aim to identify research gaps, our review is restricted to studies published in the last 9- to 15-years. Moreover, searching, conducting full-text reviews, and mapping the evidence is labor intensive, making it a challenge to keep up with new literature, which is why we limited expanded search terms in the updated search. Advancements in machine learning methods may help with selecting, tagging, and extracting data. However, as our CVD Environmental Health Disparities Tool requires full-text reviews and streamlined data extraction, usually involving careful review of data tables, the current machine learning methods are not practical to update the Tool in the near future. As noted previously, we did not evaluate study quality, and our summary of findings for special topics is based on reported results. Study quality assessments should be evaluation-specific, i.e., consider issues related to the given exposure and outcome. 

## Conclusions

Globally and in the United States, CVD is a leading cause of death and loss of health. But its burden is higher on people of certain races/ethnicity or income status. Approximately 80% of CVD is preventable [216], and identification of environmental contributors can help improve health outcomes, including equal health quality. Our CVD Environmental Health Disparities Tool of six SEMs—visualizing the study characteristics of over 6.900 primary epidemiological studies, almost 1,800 primary studies in DAP—can be used as a resource to identify research cases on the causes of environmental health disparities and to develop solutions. While there are many reviews on environmental exposures or PSS and specific CVD outcomes, we are not aware of any comprehensive systematic evidence mapping that integrates these factors across all CVD outcomes. Our tool includes 35 environmental exposures, 13 PSS, and > 30 CVD outcomes in 9 populations (8 DAPs + general). In this report, we used the tools to identify data gaps for specific environmental exposures, PSS, CVD outcomes, and specific DAP groups.

To illustrate how our CVD Environmental Health Disparities Tool can be used to facilitate environmental health disparities research and promote environmental fairness, we further analyzed several topics as exemplars (i.e., noise pollution, heat/cold stress, discrimination, AL) to identify data gaps and research recommendations for those topics (Table 5).

Much of the research we reviewed reported on established environmental causes of CVD. More studies are needed for some everyday exposures, such as environmental pollutants and consumer and personal care products. Among the many studies of established environmental causes of CVD (including noise and climate extremes), few are available that included DAPs. The contribution of these exposures to CVD health disparities is unknown, although there is a growing body of evidence suggesting an association between discrimination and several CVD outcomes. Very few studies evaluated the interaction between PSS and environmental exposures and CVD risk. The potential impact of these interactions needs to be better understood to determine whether current regulations protect DAPs from CVD risk (e.g., environmental health disparities can result from higher exposure and greater susceptibility to a given exposure level). We hope our systematic evidence mapping and CVD Environmental Health Disparities Tool will stimulate research, systematic reviews, and interventions to promote equal health quality for all.

## Supplementary Information

Supplementary Material 1.

## Data Availability

The data generated and analyzed during the review are available in the CVD Environmental Health Disparities Tool on Tableau 10.22427/NTP-DATA-500-102-001-000-9.

## Abbreviations

ACE: Adverse childhood experience
AL: Allostatic load
CAD: Coronary artery disease
CVD: Cardiovascular disease
DAP: Disproportionately affected population
DTT: Division of Translational Toxicology
dB: Decibel
EDS: Everyday Discrimination Scale
EPA: U.S. Environmental Protection Agency
EOD: Experiences of Discrimination
HAWC: Health Assessment Workspace Collaborative
HIC: High-income country
LMIC: Low- and middle-income country
MI: Myocardial infarction
NIEHS: National Institute of Environmental Health Sciences
PAD: Pulmonary artery disease
PECO: Population, exposure, comparator, and outcome
PRISMA: Preferred Reporting Items for Systematic Reviews and Meta-Analyses
PSS: Psychosocial stressors
SEM: Systematic evidence map
SES: Socioeconomic status
SRE: Schedule of Racist Events

## Glossary

Allostatic load: An accumulation of physiological perturbations – measured by a collection of biomarkers, including cardiovascular biomarkers – caused by repeated and chronic stress and adaptation to stress.
Cardiovascular disease (CVD) outcomes: Outcomes include biomarkers, subclinical/precursor effects, diseases, and clinical events related to the heart or vessels.
Disproportionately affected populations (DAP): These populations may be at higher risk of disease based on where they live, their race or ethnicity, sexual orientation, or socioeconomic status.
Environmental exposures: These include factors (chemical, physical, biological) in the physical environment (indoor, outdoor, work), consumer products, and personal behaviors (e.g., diet, tobacco smoking, and alcohol use).
Psychosocial stressors (PSS): Perceived or existing threats that create an intense level of stress that may contribute to disease or behavior changes.
Systematic Evidence Map (SEM): Interactive database that visualizes and characterizes the type and extent of research published in the literature for a specific topic.
Weathering Hypothesis: Chronic exposure to economic and social disadvantages results in an accelerated decline in health outcomes.
