# Supplementary material for: CVD Environmental Health Disparities Tool: systematic evidence mapping of psychosocial stressors, environmental exposures, and cardiovascular diseases to inform disparities research and action
Source: Environ Health. 2026 Feb 26;25:28. doi: 10.1186/s12940-026-01269-9 (PMC13059291; doi:10.1186/s12940-026-01269-9)
Supplement: Supplementary file 1 — Supplementary Material 1. [file 12940_2026_1269_MOESM1_ESM.docx]

Supplemental Material

**CVD Environmental Health Disparities Tool: Systematic Evidence Mapping of Psychosocial Stressors, Environmental Exposures, and Cardiovascular Diseases to Inform Disparities Research and Action**

Ruth M. Lunn, Katherine R. Helmick, Grace Cooney, Melissa Polansky, Samantha J. Snow, Wren Tracy, Darlene Dixon

**Table of Contents**

Literature Search

Table S1. Literature Search Information

Table S2. PubMed Literature Search Terms for Environmental Exposures

Table S3. PubMed Literature Search Terms for Psychosocial Stressors

Table S4. PubMed Literature Search Terms for Human Epidemiology

Table S5. PubMed Literature Search Terms Cardiovascular Disease Outcomes

Table S6. PubMed Literature Search Terms for Disproportionately Affected Populations

Table S7. PubMed Literature Search Terms for Targeted Environmental Exposures and Psychosocial Stressors

Literature Screening

Table S8. Inclusion and Exclusion Criteria for Title and Abstract Screening

Table S9. Inclusion and Exclusion Criteria for Full-text Screening

Table S10. Tagging Criteria for Full-text Screening

Supplemental Analysis

Table S11. Summary of Epidemiological Studies Examining the Relationship Between Discrimination and CVD Outcomes

References

Literature Search

Table S1. Literature Search Information

| Search Term Combination | Total Reference Count | | Search Dates | Comments |
| --- | --- | --- | --- | --- |
| **Main Literature Searches** |  | |  |  |
| (ENV Search Terms*^a^*) AND (Epi Primary Articles Search Terms) AND (CVD Search Terms*^b^*) | 26,688 | | 1/1/2017*^c^*–1/27/2025 | Searches run 3/19/2020, 4/11/2023, 10/9/2023, and 1/27/2025. |
| (ENV Search Terms*^a^*) AND (Epi Reviews Search Terms) AND (CVD Search Terms*^b^*) | 4,826 | | 1/1/2015*^d^*–1/27/2025 | Searches run 3/19/2020, 4/11/2023, 10/9/2023, and 1/27/2025. |
| (ENV Search Terms*^a^*) AND (Epi All Literature Search Terms) AND (CVD Search Terms*^b^*) AND (DAP Search Terms*^f^*) | 3,996 | | 1/1/2010*^e^*–1/27/2025 | Searches run 3/19/2020, 4/11/2023, 10/9/2023, and 1/27/2025. |
| (PSS Search Terms*^g^*) AND (Epi Primary Articles Search Terms) AND (CVD Search Terms*^b^*) AND (DAP Search Terms*^c^*) | 31,927 | | 1/1/2017*^c^*–1/27/2025 | Searches run 3/19/2020, 4/11/2023, 10/9/2023, and 1/27/2025. |
| (PSS Search Terms*^g^*) AND (Epi Reviews Search Terms) AND (CVD Search Terms*^b^*) AND (DAP Search Terms*^f^*) | 3,375 | | 1/1/2015*^d^*–1/27/2025 | Searches run 3/19/2020, 4/11/2023, 10/9/2023, and 1/27/2025. |
| (ENV Search Terms*^a^*) AND (PSS Search Terms*^g^*) AND (Epi All Literature Search Terms) AND (CVD Search Terms*^b^*) AND (DAP Search Terms*^f^*) | 3,101 | | 1/1/2010*^e^*–1/27/2025 | Searches run 3/19/2020, 4/11/2023, 10/9/2023, and 1/27/2025. |
| **Additional Targeted Searches for Topics of Interest** | |  |  |  |
| (AL Search Terms) AND (Epi/Animal Models All Literature Search Terms) | 2,294 | | No limit*^h^*–1/27/2025 | Searches run 11/12/2020, 4/22/2023, 10/9/2023, and 1/27/2025. |
| (SQ Search Terms) AND (AL Search Terms) AND (Epi/Animal Models All Literature Search Terms) | 28 | | 1/1/2010–1/27/2025 | Searches run 05/20/2022, 4/11/2023, 10/9/2023, and 1/27/2025. |
| (DISC Search Terms) AND (Epi Primary Articles Search Terms) AND (CVD Search Terms) | 1,600 | | 1/1/2020–1/27/2025 | Searches run 05/20/2022, 4/11/2023, 10/9/2023, and 1/27/2025. |
| ([H&C Search Terms] OR [NP Search Terms] OR [SW Search Terms] OR [WF Search Terms]) AND (Epi All Literature Search Terms) AND (CVD Search Terms) | 4,693 | | 1/1/2010–1/27/2025 | Searches run 4/11/2023, 10/9/2023, and 1/27/2025. |

Abbreviations: AL, allostatic load search terms (see Table S7); CVD, cardiovascular disease outcomes search terms (see Table S5); DAP, disproportionately affected populations search terms (see Table S6); DISC, discrimination search terms (see Table S6); ENV, environmental exposures search terms (see Table S2); Epi, human epidemiology search terms (see Table S4); H&C, heat and cold search terms (see Table S7); HT, hypertension search terms (see Table S5; NP, noise pollution search terms (see Table S7); Preg. HT, pregnancy-induced hypertension search terms (see Table S5); PSS, psychosocial stressors search terms (see Table S3); SQ, sleep quality search terms (see Table S7); SW, shift work search terms (see Table S7); WF, wildfire search terms (see Table S7).

*^a^*Specific air pollution and diet terms were removed from the ENV search terms for all literature search updates run in 2023 and onwards. Air pollution and diet were identified as established risk factors for CVD and were removed from the search terms to decrease the high volume of search results.

*^b^*The searches for cardiovascular disease outcomes, hypertension outcomes, and pregnancy-induced hypertension outcomes were originally conducted as separate searches but later combined into one search.

*^c^*To help reduce the high volume of search results, the search date for primary articles was limited to 2017.

*^d^*To help reduce the high volume of search results, the search date for reviews was limited to 2015.

*^e^*To help reduce the high volume of search results, the search date for all literature on DAPs was limited to 2010.

*^f^*Specific socioeconomic status terms were removed from the DAP search terms for all literature search updates run in 2023 and onwards. Socioeconomic status was identified as an established risk factor for CVD and was removed from the search terms to decrease the high volume of search results.

*^g^*Specific socioeconomic status and education level terms were removed from the PSS search terms for all literature search updates run in 2023 and onwards. Socioeconomic status and education level were identified as established risk factors for CVD and were removed from the search terms to decrease the high volume of search results.

*^h^*The body of allostatic load literature was small so no date limitations were implemented.

Table S2. PubMed Literature Search Terms for Environmental Exposures

| Topic | Search Parameters |
| --- | --- |
| Environmental Exposures | "Environmental Pollution"[Mesh] OR "Environmental Pollutants" [Pharmacological Action] OR "Toxic Actions"[Mesh] OR "Environmental Health"[Mesh] OR "Disorders of Environmental Origin"[Mesh] OR "Ecotoxicology"[Mesh] OR "Carcinogens, Environmental" [Pharmacological Action] OR "Hazardous Substances" [Pharmacological Action] OR "Neurotoxins" [Pharmacological Action] OR "Noxae" [Pharmacological Action] OR "Mutagens" [Pharmacological Action] OR "Teratogens" [Pharmacological Action] OR "Soil Pollutants" [Pharmacological Action] OR "Water Pollutants, Chemical" [Pharmacological Action] OR "Radioactive Pollutants"[Mesh] OR Environmental-agent*[tiab] OR environmental-chemical*[tiab] OR environmental-compound*[tiab] OR environmental-contaminant*[tiab] OR environmental-determinant*[tiab] OR environmental-estrogen*[tiab] OR environmental-exposure*[tiab] OR carcinogen*[tiab] OR teratogen*[tiab] OR mutagen*[tiab] OR pollut*[tiab] OR cardiotox*[tiab] OR ecotox*[tiab] OR toxicant*[tiab] OR toxin*[tiab] OR chemical-compound*[tiab] OR chemical-exposure*[tiab] OR chemical-mixture*[tiab] OR chemical-product*[tiab] OR chemical-substance*[tiab] OR chemical-hazard*[tiab] OR synthetic-chemical*[tiab] OR hazardous-compound*[tiab] OR hazardous-exposure*[tiab] OR hazardous-mixture*[tiab] OR hazardous-material* OR hazardous-product*[tiab] OR hazardous-substance*[tiab] OR industrial-compound*[tiab] OR industrial-chemical*[tiab] OR biohazard*[tiab] OR **"Particulate Matter"[Mesh] OR "Vehicle Emissions"[Mesh] OR "Fossil Fuels"[Mesh] OR “Air Pollutants" [Pharmacological Action] OR "Air Pollutants, Occupational" [Pharmacological Action] OR "Volatile Organic Compounds"[Mesh] OR "Heating"[Mesh] OR "Cooking"[Mesh] OR "Dust"[Mesh:NoExp] OR "Radon"[Mesh] OR particulate-matter[tiab] OR smog[tiab] OR soot[tiab] OR PM2.5[tiab] OR "PM(2.5)"[tiab] OR PM10[tiab] OR "PM(10)"[tiab] OR carbon-black[tiab] OR black-carbon[tiab] OR elemental-carbon[tiab] OR ((air[tiab] OR airborne[tiab] OR coarse[tiab] OR ultrafine[tiab] OR fine[tiab]) AND (particle*[tiab] OR particulate*[tiab])) OR ((vehicle[tiab] OR vehicles[tiab] OR vehicular[tiab] OR auto[tiab] OR automobile[tiab] OR motor*[tiab] OR bus[tiab] OR buses[tiab] OR car[tiab] OR truck*[tiab] OR taxi[tiab] OR taxis[tiab] OR motorcycle*[tiab] OR engine*[tiab] OR traffic[tiab] OR road*[tiab] OR street*[tiab] OR highway*[tiab] OR interstate*[tiab] OR transport*[tiab] OR factory[tiab] OR factories[tiab] OR industr*[tiab] OR manufactur*[tiab]) AND (emission*[tiab] OR exhaust[tiab] OR fume*[tiab])) OR sulfur-dioxide[tiab] OR S02[tiab] OR ozone[tiab] OR O3[tiab] OR hydrogen-sulfide[tiab] OR H2S[tiab] OR carbon-monoxide[tiab] OR nitric-oxide[tiab] OR nitrogen-oxide[tiab] OR nitrogen-oxides[tiab] OR nitrogen-dioxide[tiab] OR NOx[tiab] OR "NO(x)"[tiab] OR NO2[tiab] OR volatile-organic-compound*[tiab] OR VOCs[tiab] OR gasoline*[tiab] OR diesel[tiab] OR petrol*[tiab] OR burn-pit*[tiab] OR ((wood[mesh] OR wood[tiab] OR firewood[tiab] OR biomass* OR charcoal[tiab] OR fuel[tiab] OR fuels[tiab] OR gas[tiab] OR gasoline[tiab] OR kerosene[tiab] OR dung[tiab] OR manure[tiab]) AND (smoke[mesh] OR combust*[tiab] OR burn*[tiab] OR burning[tiab])) OR cooker*[tiab] OR cooking[tiab] OR stove*[tiab] OR oven*[tiab] OR cookstove*[tiab] OR cook-stove*[tiab] OR woodstove*[tiab] OR heater*[tiab] OR fireplace*[tiab] OR woodsmoke[tiab] OR dust[tiab] OR radon[tiab] OR** "Allergens"[Mesh] OR "Pollen"[Mesh:NoExp] OR "Dander"[Mesh] OR "Mites"[Mesh] OR allerg*[tiab] OR aeroallergen*[tiab] OR dander[tiab] OR mite[tiab] OR mites[tiab] OR cockroach*[tiab] OR pollen[tiab] OR housedust[tiab] OR **"Dietary Exposure"[Mesh] OR "Food Contamination"[Mesh:NoExp] OR "Aflatoxins"[Mesh] OR "Fluorides"[Mesh] OR "Mycotoxins"[Mesh] OR "Isoflavones"[Mesh] OR "Polybrominated Biphenyls"[Mesh] OR "Trace Elements" [Pharmacological Action] OR dietary exposure*[tiab] OR ((food[tiab] OR drinking water[tiab]) AND (contamination[tiab] OR contaminant*[tiab])) OR selenium[tiab] OR mycotoxin*[tiab] OR isoflavone*[tiab] OR isoflavonoid*[tiab] OR genistein[tiab] OR phytoestrogen*[tiab] OR phytochemical*[tiab] OR trace elements[tiab] OR plasma metal[tiab] OR plasma metals[tiab] OR** "Dioxins and Dioxin-like Compounds"[Mesh] OR dioxane*[tiab] OR dioxin*[tiab] OR furan[tiab] OR furans[tiab] OR dibenzofuran*[tiab] OR heptachlorodibenzodioxin[tiab] OR HpCDD[tiab] OR HCDD[tiab] OR hexachlorodibenzodioxin[tiab] OR HxCDD[tiab] OR PeCDD[tiab] OR dibenzodioxin*[tiab] OR PCDD[tiab] OR Tetrachlorodibenzodioxin[tiab] OR TCDD[tiab] OR Heptachlorodibenzofuran[tiab] OR Hexachlorodibenzofuran[tiab] OR Octachlorodibenzofuran[tiab] OR pentachlorodibenzofuran[tiab] OR tetrachlorodibenzofuran[tiab] OR TCDF[tiab] OR (endocrine[tiab] AND disrupt*[tiab]) OR bisphenol[tiab] OR BPA[tiab] OR dinitrobisphenol[tiab] OR tetrabromobisphenol[tiab] OR "2,2-bis(4-hydroxyphenyl)propane"[tiab] OR 4-tert-octylphenol[tiab] OR p-tert-octylphenol[tiab] OR 4-tertiary-octylphenol[tiab] OR "p-(1,1,3,3-tetramethylbutyl)-phenol"[tiab] OR triclocarban[tiab] OR trichlorocarbanilide[tiab] OR trichlorcarban[tiab] OR Triclosan[tiab] OR butylparaben[tiab] OR hydroxybenzoate[tiab] OR ethylparaben[tiab] OR methylparaben[tiab] OR polychlorinated biphenyl*[tiab] OR PCB* [tiab] OR Tetrachlorobiphenyl[tiab] OR pentachlorobiphenyl[tiab] OR hexachlorobiphenyl[tiab] OR Heptachlorobiphenyl[tiab] OR trichlorobiphenyl[tiab] OR Octachlorobiphenyl[tiab] OR Nonachlorobiphenyl[tiab] OR Decachlorobiphenyl[tiab] OR diethylstilbestrol[tiab] OR stilbestrol[tiab] OR stilbene-estrogen[tiab] OR xenoestrogen*[tiab] OR tributylin[tiab] OR nonylphenol[tiab] OR ethynylestradiol*[tiab] OR "Electronic Waste"[Mesh] OR electronic-waste*[tiab] OR electronics-waste* OR electronic-scrap*[tiab] OR Electronics-recycling[tiab] OR e-scrap*[tiab] OR e-waste*[tiab] OR EWRSs[tiab] OR Scrap-computer*[tiab] OR waste-electronic*[tiab] OR WEEE[tiab] OR ((electronic*[tiab] OR cell phone[tiab] OR cellular phone[tiab] OR mobile phone*[tiab] OR computer[tiab] OR television*[tiab] OR "TV"[tiab] OR "TVs"[tiab] OR laptop*[tiab] OR tablet*[tiab] OR mobile-device*[tiab] OR MP3-player*[tiab] OR CD-player*[tiab] OR DVD-player*[tiab] OR sound-system[tiab] OR home-entertainment*[tiab] OR camera*[tiab] OR game-console*[tiab] OR calculator*[tiab] OR circuit-board*[tiab] OR mother-board*[tiab] OR motherboard*[tiab] OR crt-funnel-glass[tiab] OR liquid-crystal[tiab] OR amoled[tiab] OR LCD[tiab] OR cathode-ray-tube*[tiab] OR battery[tiab] OR batteries[tiab]) AND ("Recycling"[Mesh] OR "Refuse Disposal"[Mesh] OR "Solid Waste"[Mesh] OR waste[tiab] OR recycle*[tiab] OR disposal*[tiab] OR scrap[tiab] OR scrapped[tiab] OR scraps[tiab])) OR "Flame Retardants"[Mesh] OR "Flame Retardants" [Pharmacological Action] OR "Halogenated Diphenyl Ethers"[Mesh] OR "tris(2,3-dibromopropyl)phosphate" [Supplementary Concept] OR "bis(1-chloro-2-propyl)phosphate" [Supplementary Concept] OR "bis(2-butoxyethyl)phosphate" [Supplementary Concept] OR fire-retard*[tiab] OR flame-retard*[tiab] OR fire-proofing[tiab] OR ((halogenated[tiab] OR chlorinated[tiab] OR brominated[tiab] OR polybrominated[tiab]) AND (diphenyl-ether*[tiab])) OR PBDE*[tiab] OR PCDE*[tiab] OR BDE*[tiab] OR tribrominated diphenyl ether[tiab] OR tribromodiphenyl ether[tiab] OR tetrabromodiphenyl ether[tiab] OR brominated diphenyl ether[tiab] OR tetraBDE[tiab] OR tetrabrominated diphenyl ether[tiab] OR pentabromodiphenyl ether[tiab] OR pentaBDE[tiab] OR hexabromodiphenyl ether[tiab] OR hexabromodiphenyl[tiab] OR hexaBDE[tiab] OR heptabromodiphenyl ether[tiab] OR HeptaBDE[tiab] OR hexabromocyclododecane[tiab] OR HBCD[tiab] OR 345-HBB[tiab] OR 245-HBB[tiab] OR PBB-153[tiab] OR PBB153[tiab] OR asbestos[tiab] OR TDBPP[tiab] OR "Metals, Heavy"[Mesh] OR "Heavy Metal Poisoning"[Mesh] OR "Heavy Metal Poisoning, Nervous System"[Mesh] OR "Methylmercury Compounds"[Mesh] OR heavy-metal*[tiab] OR toxic-metal*[tiab] OR synthetic-metal*[tiab] OR arsenic[tiab] OR arsenical*[tiab] OR arsenite*[tiab] OR arsenate*[tiab] OR ((Titanium[tiab] OR Vanadium[tiab] OR Chromium[tiab] OR Manganese[tiab] OR Iron[tiab] OR Cobalt[tiab] OR Nickel[tiab] OR Copper[tiab] OR Zinc[tiab] OR Gallium[tiab] OR Germanium[tiab] OR Zirconium[tiab] OR Niobium[tiab] OR Molybdenum[tiab] OR Technetium[tiab] OR Ruthenium[tiab] OR Rhodium[tiab] OR Palladium[tiab] OR Silver[tiab] OR Cadmium[tiab] OR Indium[tiab] OR Tin[tiab] OR Tellurium[tiab] OR Lutetium[tiab] OR Hafnium[tiab] OR Tantalum[tiab] OR Tungsten[tiab] OR Rhenium[tiab] OR Osmium[tiab] OR Iridium[tiab] OR Platinum[tiab] OR Gold[tiab] OR Mercury[tiab] OR Methylmercury[tiab] OR Thallium[tiab] OR Bismuth[tiab] OR Polonium[tiab] OR Astatine[tiab] OR Lanthanum[tiab] OR Cerium[tiab] OR Praseodymium[tiab] OR Neodymium[tiab] OR Promethium[tiab] OR Samarium[tiab] OR Europium[tiab] OR Gadolinium[tiab] OR Terbium[tiab] OR Dysprosium[tiab] OR Holmium[tiab] OR Erbium[tiab] OR Thulium[tiab] OR Ytterbium[tiab] OR Actinium[tiab] OR Thorium[tiab] OR Protactinium[tiab] OR Uranium[tiab] OR Neptunium[tiab] OR Plutonium[tiab] OR Americium[tiab] OR Curium[tiab] OR Berkelium[tiab] OR Californium[tiab] OR Einsteinium[tiab] OR Fermium[tiab] OR Radium[tiab] OR Bohrium[tiab] OR rare earth[tiab]) AND (poison*[tiab] OR exposure*[tiab] OR exposed[tiab] OR contaminat*[tiab] OR dietary[tiab] OR blood[tiab] OR serum[tiab] OR urine[tiab] OR urinary[tiab])) OR blood-lead[tiab] OR (lead[tiab] AND PB[tiab]) OR lead exposure*[tiab] OR dietary lead[tiab] OR lead poisoning[tiab] OR lead-level*[tiab] OR "Household Products"[Mesh] OR "Cosmetics"[Mesh] OR personal product*[tiab] OR cosmetics[tiab] OR cosmetic product*[tiab] OR care product*[tiab] OR consumer product*[tiab] OR commercial product*[tiab] OR cleaning product*[tiab] OR household product*[tiab] OR consumer goods[tiab] OR makeup[tiab] OR make-up[tiab] OR toys[tiab] OR plastic container*[tiab] OR food container*[tiab] OR "Polycyclic Aromatic Hydrocarbons"[Mesh] OR "Benzene"[Mesh] OR polycyclic-aromatic-hydrocarbons[tiab] OR PAHs[tiab] OR fumonisin[tiab] OR benzopyrene[tiab] OR benzo-a-pyrene[tiab] OR "3,4-benzopyrene"[tiab] OR hydroxypyrene[tiab] OR benzene[tiab] OR toluene[tiab] OR xylene[tiab] OR "Pesticides" [Pharmacological Action] OR "Herbicides" [Pharmacological Action] OR "Insecticides" [Pharmacological Action] OR "Rodenticides" [Pharmacological Action] OR Pesticid*[tiab] OR fungicide*[tiab] OR herbicide*[tiab] OR insecticide*[tiab] OR insect-repellent*[tiab] OR rodenticide*[tiab] OR acaricide*[tiab] OR algicide*[tiab] OR biopesticide*[tiab] OR fumigant*[tiab] OR molluscicide*[tiab] OR nematicide*[tiab] OR weed-killer*[tiab] OR pest control[tiab] OR organochlorin*[tiab] OR organic-chlorine[tiab] OR chlorinated-hydrocarbon*[tiab] OR aldrin[tiab] OR chlordan[tiab] OR chlordane[tiab] OR chlordecone[tiab] OR chlorobenzene*[tiab] OR chlorofluorocarbon*[tiab] OR dichlorophenyl-dichloroethylene[tiab] OR dichlorodiphenyltrichloroethane*[tiab] OR DDT[tiab] OR Dichlorodiphenyldichloroethylene[tiab] OR DDE[tiab] OR dieldrin[tiab] OR endrin[tiab] OR heptachlor[tiab] OR hexachlorobenzene[tiab] OR hexachlorocyclohexane[tiab] OR alpha-HCH[tiab] OR beta-HCH[tiab] OR lindane[tiab] OR hexachlorocyclohexane[tiab] OR methoxychlor[tiab] OR mirex[tiab] OR polychlorinated-biphenyl*[tiab] OR "polychlorinated biphenyls"[tiab] OR polychlorobiphenyl[tiab] OR PCBs[tiab] OR tetrachloroethylene[tiab] OR trichloroethane*[tiab] OR vinyl-chloride[tiab] OR agent-orange[tiab] OR Amitraz[tiab] OR atrazine[tiab] OR avermectin[tiab] OR captan[tiab] OR carbaryl[tiab] OR carbofuran[tiab] OR chlorfenvinphos[tiab] OR chlorpyrifos[tiab] OR coumaphos[tiab] OR deet[tiab] OR "N,N-diethyltoluamide"[tiab] OR diazinon[tiab] OR dichlorvos[tiab] OR "dimethyl phthalate"[tiab] OR endosulfan*[tiab] OR linalool[tiab] OR malathion[tiab] OR paraquat[tiab] OR parathion[tiab] OR pentachlorobenzene[tiab] OR PeCB[tiab] OR pentachlorophenol[tiab] OR permethrin[tiab] OR pyrethrin*[tiab] OR rotenone[tiab] OR vinclozolin[tiab] OR omethoate[tiab] OR dichlorophenol[tiab] OR “2,5-DCP”[tiab] OR dichlorophenol[tiab] OR “2,4-DCP”[tiab] OR trichlorophenol[tiab] OR “2,4,5-TCP”[tiab] OR trichlorophenol[tiab] OR “2,4,6-TCP”[tiab] OR "Fluorocarbons/adverse effects"[Mesh] OR "perfluorododecanoic acid"[Supplementary Concept] OR PFASs[tiab] OR PFOS[tiab] OR perfluoroalkyl[tiab] OR polyfluoroalkyl[tiab] OR perfluorobutanesulfonic[tiab] OR perfluorodecanoic[tiab] OR perfluoro-n-decanoic[tiab] OR nonadecafluoro-n-decanoic[tiab] OR perfluorododecanoic[tiab] OR PFDoA[tiab] OR perfluoroheptanoic[tiab] OR PFHpA[tiab] OR perfluorohexanesulfonic[tiab] OR PFHS[tiab] OR perfluorohexanesulfonate[tiab] OR perfluorononanoic[tiab] OR perfluorooctanoic[tiab] OR pentadecafluorooctanoic[tiab] OR perfluorooctanoyl-chloride[tiab] OR PFOA[tiab] OR sodium-perfluorooctanoate[tiab] OR perfluorooctanoate[tiab] OR ammonium-perfluorooctanoate[tiab] OR "APFO"[tiab] OR perfluorooctane-sulfonic[tiab] OR perfluorooctanesulfonic[tiab] OR PFOSA[tiab] OR perfluorooctanyl-sulfonate[tiab] OR Perfluorooctanesulfonate[tiab] OR perfluorooctane-sulfonate[tiab] OR perfluorooctanesulfonamide[tiab] OR perfluoroundecanoic[tiab] OR "Phthalic Acids"[Mesh] OR "Plasticizers"[Mesh] OR "Plasticizers" [Pharmacological Action] OR Phthalate*[tiab] OR butylbenzenesulfonamide[tiab] OR plasticizers[tiab] OR MEOHP[tiab] OR MEHP[tiab] OR DEHP[tiab] OR “Bis(2-ethylhexyl)phthalate”[tiab] OR "Solvents" [Pharmacological Action] OR solvents[tiab] OR organic-solvent[tiab] OR "2-bromopropane"[tiab] OR "2-propanol"[tiab] OR acetone[tiab] OR tetrachloroethylene[tiab] OR toluene[tiab] OR trichloroethylene |

Search terms in bold text were removed from the search updates run in 2023 to reduce the large number of search results captured. Air pollution and diet search terms were removed from the environmental exposures search string as these were identified as established risk factors of cardiovascular disease.

Table S3. PubMed Literature Search Terms for Psychosocial Stressors

| Topic | Search Parameters |
| --- | --- |
| Psychosocial Stressors | "Social Discrimination"[Mesh] OR "Social Segregation"[Mesh] OR ((race[tiab] OR racial*[tiab] OR ethnic[tiab] OR social[tiab] OR perceived[tiab]) AND (discriminat*[tiab] OR bias[tiab] OR segregation[tiab] OR inequity[tiab] OR inequities[tiab] OR inequality[tiab] OR inequalities[tiab])) OR prejudice[tiab] OR racism[tiab] OR racist[tiab] OR racial-isolation[tiab] OR racial-composition[tiab] OR "Built Environment"[Mesh] OR "Housing"[Mesh] OR "Residence Characteristics"[Mesh] OR (residential[tiab] AND (area[tiab] OR block[tiab] OR street*[tiab] OR district*[tiab] OR subdivision[tiab] OR environment[tiab] OR characteristics[tiab] OR fetures[tiab])) OR housing[tiab] OR neighborhood*[tiab] OR neighbourhood*[tiab] OR slum[tiab] OR slums[tiab] OR census-tract*[tiab] OR census-block*[tiab] OR zipcode[tiab] OR area level[tiab] OR Geocode*[tiab] OR geo-linked[tiab] OR green space*[tiab] OR greenspace*[tiab] OR residential greenness[tiab] OR community garden*[tiab] OR traffic-proximity[tiab] OR traffic exposure[tiab] OR food access[tiab] OR food desert*[tiab] OR walkability[tiab] OR walkable[tiab] OR unwalkable[tiab] OR residential mobility[tiab] OR residential-preference*[tiab] OR residential-composition[tiab] OR residential-isolation[tiab] OR residential segregation[tiab] OR "Sociological Factors"[Mesh] OR "Social Behavior"[Mesh] OR Psychosocial[tiab] OR psychobiological[tiab] OR psycho-biological[tiab] OR psychophysiological [tiab] psychophysiological[tiab] OR social environment[tiab] OR social network*[tiab] OR social contact*[tiab] OR social factors[tiab] OR sociological factors[tiab] OR sociocultural[tiab] OR socio-cultural[tiab] OR Life stress[tiab] OR psychological stress[tiab] OR emotional stress[tiab] OR social stress[tiab] OR daily stress OR perceived stress[tiab] OR hostility[tiab] OR anger[tiab] OR worry[tiab] OR anxiety[tiab] OR distress[tiab] OR depression[tiab] OR hopelessness[tiab] OR and self-respect[tiab] OR self-confidence[tiab] OR loneliness[tiab] OR optimism[tiab] OR **"Socioeconomic Factors"[Mesh] OR "Homeless Persons"[Mesh] OR "Transients and Migrants"[Mesh] OR "Working Poor"[Mesh] OR "Health Status Disparities"[Mesh] OR "Healthcare Disparities"[Mesh] OR "Social Determinants of Health"[Mesh] OR "Health Equity"[Mesh] OR "Occupational Stress"[Mesh] OR "Rural Population"[Mesh] OR "Rural Health"[Mesh] OR "Urban Health"[Mesh] OR "Urban Population"[Mesh] OR Socio-economic*[tiab] OR socioeconomic*[tiab] OR social-disadvantage*[tiab] OR socially disadvantaged[tiab] OR economic disadvantage*[tiab] OR economically disadvantaged[tiab] OR social class[tiab] OR social mobility[tiab] OR poverty[tiab] OR homeless*[tiab] OR depravation[tiab] OR deprived[tiab] OR urban poor[tiab] OR urban population*[tiab] OR urban health[tiab] OR inner city[tiab] OR rural health[tiab] OR rural population*[tiab] OR working poor[tiab] OR income*[tiab] OR salary[tiab] OR salaries[tiab] OR financial strain[tiab] OR financial debt*[tiab] OR job loss[tiab] OR unemployed[tiab] OR unemployment[tiab] OR employment[tiab] OR employed[tiab] OR affordability[tiab] OR social risk factor*[tiab] OR education[tiab] OR literacy[tiab] OR diploma[tiab] OR job stability[tiab] OR career stability[tiab] OR work stability[tiab] OR professional stability[tiab] OR job instability[tiab] OR career instability[tiab] OR work instability[tiab] OR professional instability[tiab] OR blue-collar[tiab] OR white-collar[tiab] OR disparities[tiab] OR disparity[tiab] OR social determinant*[tiab] OR health equity[tiab] OR health inequalities[tiab] OR health inequality[tiab] OR social inequality[tiab] OR health justice[tiab] OR health injustices[tiab] OR sociodemographic[tiab]** |

Search terms in bold text were removed from the search updates run in 2023 to reduce the large number of search results captured. Socioeconomic status and education level, topics identified as established risk factors for cardiovascular disease, were removed from the psychosocial stressor search terms for the 2023 search updates.

Table S4. PubMed Literature Search Terms for Human Epidemiology

| Topic | Search Parameters |
| --- | --- |
| Human Epidemiology All Literature | ("Epidemiological Monitoring"[Mesh] OR "Epidemiologic Studies"[Mesh] OR "epidemiology"[sh] OR "Meta-Analysis"[pt] OR "Review" [Publication Type] OR "Systematic Review" [Publication Type] OR "Randomized Controlled Trial"[pt] OR ("Environmental Exposure"[Mesh] NOT "Environmental Monitoring"[Mesh]) OR morbidity[tiab] OR mortality[tiab] OR Epidemio*[tiab] OR cohort*[tiab] OR case-control[tiab] OR correlation stud*[tiab] OR retrospective[tiab] OR follow-up[tiab] OR longitudinal[tiab] OR prospective[tiab] OR retrospective[tiab] OR cross-sectional[tiab] OR seroepidemio*[tiab] OR case-referent[tiab] OR record-link*[tiab] OR ecologic stud*[tiab] OR aggregate stud*[tiab] OR observational stud*[tiab] OR randomized controlled trial[tiab] OR randomised controlled trial[tiab] OR controlled clinical trial[tiab] OR randomized[tiab] OR randomly[tiab] OR placebo[tiab] OR trial[ti] OR metaanalysis[tiab] OR metaanalyses[tiab] OR meta-analysis[tiab] OR meta-analyses[tiab]) NOT ("Animals"[Mesh] NOT "Humans"[Mesh]) |
| Human Epidemiology Primary Articles | ("Epidemiological Monitoring"[Mesh] OR "Epidemiologic Studies"[Mesh] OR "epidemiology"[sh] OR "Randomized Controlled Trial"[pt] OR ("Environmental Exposure"[Mesh] NOT "Environmental Monitoring"[Mesh]) OR morbidity[tiab] OR mortality[tiab] OR Epidemio*[tiab] OR cohort*[tiab] OR case-control[tiab] OR correlation stud*[tiab] OR retrospective[tiab] OR follow-up[tiab] OR longitudinal[tiab] OR prospective[tiab] OR retrospective[tiab] OR cross-sectional[tiab] OR seroepidemio*[tiab] OR case-referent[tiab] OR record-link*[tiab] OR ecologic stud*[tiab] OR aggregate stud*[tiab] OR observational stud*[tiab] OR randomized controlled trial[tiab] OR randomised controlled trial[tiab] OR controlled clinical trial[tiab] OR randomized[tiab] OR randomly[tiab] OR placebo[tiab] OR trial[ti]) NOT ("Meta-Analysis"[pt] OR "review"[pt] OR "Systematic Review" [pt] OR review[ti] OR metaanalysis[tiab] OR metaanalyses[tiab] OR meta-analysis[tiab] OR meta-analyses[tiab]) |
| Human Epidemiology Reviews | ("Epidemiological Monitoring"[Mesh] OR "Epidemiologic Studies"[Mesh] OR "epidemiology"[sh] OR "Randomized Controlled Trial"[pt] OR morbidity[tiab] OR mortality[tiab] OR Epidemio*[tiab] OR cohort*[tiab] OR case-control[tiab] OR correlation stud*[tiab] OR retrospective[tiab] OR follow-up[tiab] OR longitudinal[tiab] OR prospective[tiab] OR retrospective[tiab] OR cross-sectional[tiab] OR seroepidemio*[tiab] OR case-referent[tiab] OR record-link*[tiab] OR ecologic stud*[tiab] OR aggregate stud*[tiab] OR observational stud*[tiab] OR randomized controlled trial[tiab] OR randomised controlled trial[tiab] OR controlled clinical trial[tiab] OR randomized[tiab] OR randomly[tiab] OR placebo[tiab] OR trial[ti]) AND ("Meta-Analysis"[pt] OR "Review"[pt] OR "Systematic Review" [pt] OR review[ti] OR metaanalysis[tiab] OR case-report[tiab] OR metaanalyses[tiab] OR meta-analysis[tiab] OR meta-analyses[tiab]) |
| Human Epidemiology and Animal Models All Literature | ("Epidemiological Monitoring"[Mesh] OR "Epidemiologic Studies"[Mesh] OR "epidemiology"[sh] OR "Meta-Analysis"[pt] OR "Review" [Publication Type] OR "Systematic Review" [Publication Type] OR "Surveys and Questionnaires"[Mesh] OR "Environmental Exposure"[Mesh] OR morbidity[tiab] OR mortality[tiab] OR Epidemio*[tiab] OR cohort*[tiab] OR case-control[tiab] OR correlation stud*[tiab] OR retrospective[tiab] OR follow-up[tiab] OR longitudinal[tiab] OR prospective[tiab] OR retrospective[tiab] OR cross-sectional[tiab] OR seroepidemio*[tiab] OR case-series[tiab] OR case-referent[tiab] OR record-link*[tiab] OR ecologic stud*[tiab] OR aggregate stud*[tiab] OR observational stud*[tiab] OR survey*[tiab] OR questionnaire*[tiab]) OR ((metaanalysis[tiab] OR case-report[tiab] OR metaanalyses[tiab] OR meta-analysis[tiab]) NOT medline[sb]) |

Table S5. PubMed Literature Search Terms Cardiovascular Disease Outcomes

| Topic | Search Parameters |
| --- | --- |
| Cardiovascular Disease Outcomes | "Cardiovascular Diseases"[Mesh:NoExp] OR "atherosclerosis"[Mesh] OR “carotid artery diseases” [Mesh] OR “coronary artery disease” [Mesh] OR “myocardial infarction” [Mesh] OR “stroke” [Mesh] OR “cerebrovascular disorders” [Mesh] OR “peripheral vascular diseases” [Mesh] OR “peripheral arterial disease” [Mesh] OR Cardiovascular disease*[tiab] OR cardio-vascular disease*[tiab] OR heart disease*[tiab] OR cardiac disease*[tiab] OR coronary artery disease*[tiab] OR coronary microvascular disease[tiab] OR cerebrovascular disease*[tiab] OR vascular disease*[tiab] OR cardiovascular health[tiab] OR cardiovascular risk*[tiab] OR cardiovascular outcome*[tiab] OR cardiovascular mortality[tiab] OR cardiovascular event*[tiab] OR heart health[tiab] OR cardiac health[tiab] OR arterial stiffening[tiab] OR arterial stiffness[tiab] OR artery stiffness[tiab] OR artery calcification[tiab] OR arterial calcification[tiab] OR blood cholesterol[tiab] OR total cholesterol[tiab] OR stroke[tiab] OR (myocardi*[tiab] AND infarct*[tiab]) OR acute MI[tiab] OR Myocardial Ischemia[tiab] OR heart failure[tiab] OR cardiac failure[tiab] OR heart attack*[tiab] OR heartbeat*[tiab] OR heart beat*[tiab] OR heart rate*[tiab] OR blood pressure[tiab] OR hypertens*[tiab] OR cardiac dysrhythmia*[tiab] OR atherosclerosis[tiab] OR atherosclerot*[tiab] OR left-ventricular mass index[tiab] OR left-ventricular ejection fraction[tiab] OR intima media thickness[tiab] OR coronary revascularization[tiab] OR myocardial revascularization[tiab] OR artery stent[tiab] OR artery bypass[tiab] OR myocardial ischemia[tiab] |
| Expanded Hypertension Outcomes | "Hypertension"[Mesh] OR "Antihypertensive Agents" [Pharmacological Action] OR ace inhibitor*[tiab] OR Angiotensin Converting Enzyme Inhibitor*[tiab] OR Angiotensin II receptor blocker*[tiab] OR adrenergic inhibitor*[tiab] OR adrenergic modifier*[tiab] OR Alpha-2 Receptor Agonist*[tiab] OR alpha blocker*[tiab] OR calcium channel blocker*[tiab] OR antihypertens*[tiab] OR beta-blocker*[tiab] OR central agonist*[tiab] OR direct renin inhibitor*[tiab] OR hypertens*[tiab] OR prehypertens*[tiab] OR blood pressure[tiab] OR arterial pressure[tiab] OR systolic[tiab] OR diastolic[tiab] OR systemic vascular resistance[tiab] OR cardiac output[tiab] OR vasodilator*[tiab] |
| Expanded Pregnancy-induced Hypertension Outcomes | "Hypertension, Pregnancy-Induced"[Mesh] OR ((gestational[tiab] OR pregnancy[tiab] OR pregnant[tiab] OR prenatal[tiab] OR pre-natal[tiab]) AND (ace inhibitor*[tiab] OR Angiotensin Converting Enzyme Inhibitor*[tiab] OR Angiotensin II receptor blocker*[tiab] OR adrenergic inhibitor*[tiab] OR adrenergic modifier*[tiab] OR Alpha-2 Receptor Agonist*[tiab] OR alpha blocker*[tiab] OR calcium channel blocker*[tiab] OR antihypertens*[tiab] OR beta-blocker*[tiab] OR central agonist*[tiab] OR direct renin inhibitor*[tiab] OR hypertens*[tiab] OR prehypertens*[tiab] OR blood pressure[tiab] OR arterial pressure[tiab] OR systolic[tiab] OR diastolic[tiab] OR systemic vascular resistance[tiab] OR cardiac output[tiab] OR vasodilator*[tiab])) |

Table S6. PubMed Literature Search Terms for Disproportionately Affected Populations

| Topic | Search Parameters |
| --- | --- |
| Disproportionately Affected Populations | "Race Factors"[Mesh] OR "Minority Groups"[Mesh] OR "Minority Health"[Mesh] OR ethnic*[tiab] OR race[tiab] OR races[tiab] OR racial*[tiab] OR minorit*[tiab] OR nonwhite*[tiab] OR non-white*[tiab] OR "Social Discrimination"[Mesh] OR "Social Segregation"[Mesh] OR "Health Status Disparities"[Mesh] OR "Healthcare Disparities"[Mesh] OR ((social[tiab] OR perceived[tiab]) AND (discriminat*[tiab] OR bias[tiab] OR segregation[tiab] OR inequity[tiab] OR inequities[tiab] OR inequality[tiab] OR inequalities[tiab] OR stigma*[tiab])) OR prejudice[tiab] OR racism[tiab] OR racist[tiab] OR disparities[tiab] OR disparity[tiab] OR social determinant*[tiab] OR health equity[tiab] OR health inequalities[tiab] OR health inequality[tiab] OR social inequality[tiab] OR health justice[tiab] OR health injustices[tiab] OR social-disadvantage*[tiab] OR socially disadvantaged[tiab] OR **economic disadvantage*[tiab] OR economically disadvantaged[tiab] OR poverty[tiab] OR depravation[tiab] OR deprived[tiab] OR urban poor[tiab] OR** urban population*[tiab] OR inner city[tiab] OR rural health[tiab] OR rural population*[tiab] OR "African Americans"[Mesh] OR black*[tiab] OR African American*[tiab] OR afro-american*[tiab] OR African ancestry[tiab] OR African descent[tiab] OR Caribbean American*[tiab] OR Caribbean islanders[tiab] OR Virgin Islands[tiab] OR "Hispanic Americans"[Mesh] OR hispanic*[tiab] OR latina*[tiab] OR latino*[tiab] OR latinx[tiab] OR Mexican-american*[tiab] OR Cuban-American*[tiab] OR Tejano[tiab] OR chicano*[tiab] OR puerto-rican*[tiab] OR Puerto Rico[tiab] OR "Indians, North American"[Mesh] OR Native American*[tiab] OR American Indian*[tiab] OR Indian tribe*[tiab] OR tribal health[tiab] OR Alaskan Native*[tiab] OR Alaska Native*[tiab] OR Native Alaskan*[tiab] OR Native Hawaiian*[tiab] OR Hawaiian Native*[tiab] OR Hawaii Native*[tiab] OR ((Ak Chin[tiab] OR Cocopah[tiab] OR Yavapai[tiab] OR Havasupai[tiab] OR Hopi[tiab] OR Hualapai[tiab] OR Paiute[tiab] OR Navajo[tiab] OR Yaqui[tiab] OR Quechan[tiab] OR Pima[tiab] OR Maricopa[tiab] OR Apache[tiab] OR Tohono O’odham[tiab] OR Yavapai [tiab] OR Cahuilla[tiab] OR Alturas[tiab] OR Maidu[tiab] OR Pomo[tiab] OR Chemehuevi[tiab] OR Shoshone[tiab] OR Kumeyaay[tiab] OR Federated Indians[tiab] OR Hoopa[tiab] OR Jamul[tiab] OR Karuk[tiab] OR Luiseño[tiab] OR Cupeno[tiab] OR Lytton[tiab] OR Quechan[tiab] OR Serrano[tiab] OR Diegueño[tiab] OR Chumash[tiab] OR Kumeyaay[tiab] OR Tejon[tiab] OR United Auburn[tiab] OR Washoe[tiab] OR Wilton[tiab] OR Yocha Dehe[tiab] OR Yurok[tiab] OR Southern Ute[tiab] OR Miccosukee[tiab] OR Seminole[tiab] OR Coeur D’Alene[tiab] OR Kootenai[tiab] OR Nez Perce[tiab] OR Bannock[tiab] OR Potawatomi[tiab] OR (Sac[tiab] AND Fox[tiab]) OR Kickapoo[tiab] OR Coushatta[tiab] OR Choctaw[tiab] OR Micmac[tiab] OR Maliseet[tiab] OR Passamaquoddy[tiab] OR Penobscot[tiab] OR Wampanoag[tiab] OR Chippewa[tiab] OR Odawa[tiab] OR Sioux[tiab] OR Assiniboine[tiab] OR Blackfeet[tiab] OR Cree[tiab] OR Salish[tiab] OR Kootenai[tiab] OR Cheyenne[tiab] OR Ponca[tiab] OR Santee[tiab] OR Winnebago[tiab] OR Washoe[tiab] OR Winnemucca[tiab] OR Jicarilla OR Acoma[tiab] OR Cochiti[tiab] OR Jemez[tiab] OR Isleta[tiab] OR Laguna[tiab] OR Nambe[tiab] OR Sandia[tiab] OR Kewa[tiab] OR Zia[tiab] OR Zuni[tiab] OR Cayuga[tiab] OR Oneida[tiab] OR Onondaga[tiab] OR Mohawk[tiab] Or Seneca[tiab] OR Tonawanda[tiab] OR Tuscarora[tiab] OR Shinnecock[tiab] OR Cherokee[tiab] OR Caddo[tiab] OR Arapaho[tiab] OR Comanche[tiab] OR Shawnee[tiab] OR Kaw[tiab] OR Kiowa[tiab] OR Modoc[tiab] OR Muscogee[tiab] OR Pawnee[tiab] OR Quapaw[tiab] OR Seminole[tiab] OR Cayuga[tiab] OR Chickasaw[tiab] OR Osage[tiab] OR Wichita[tiab] OR Wyandotte[tiab] OR Coos[tiab] OR Umpqua[tiab] OR Siuslaw[tiab] OR Siletz[tiab] OR Umatilla[tiab] OR Coquille[tiab] OR Klamath[tiab] OR Narragansett[tiab] OR Catawba[tiab] OR Flandreau Santee[tiab] OR Brule[tiab] OR Oglala[tiab] OR Oyate[tiab] OR Yankton[tiab] OR Goshute[tiab] OR Shoshoni[tiab] OR Pamunkey[tiab] OR Chickahominy[tiab] OR Rappahannock[tiab] OR Monacan[tiab] OR Nansemond[tiab] OR Chehalis[tiab] OR Yakama[tiab] OR Cowlitz[tiab] OR Hoh[tiab] OR S’Klallam[tiab] OR Elwha[tiab] OR Lummi[tiab] OR Makah[tiab] OR Nisqually[tiab] OR Nooksack[tiab] OR Puyallup[tiab] OR Quinault[tiab] OR Samish[tiab] OR Shoalwater[tiab] OR Snoqualmie[tiab] OR Spokane[tiab] OR Squaxin[tiab] OR Suquamish[tiab] OR Swinomish[tiab] OR Tulalip[tiab] OR Skagit[tiab] OR Ho-Chunk[tiab] OR Menominee[tiab]) AND (Indigenous[tiab] OR reservation*[tiab] OR native*[tiab] OR tribe*[tiab] OR tribal[tiab] OR village*[tiab] OR communit*[tiab] OR nation[tiab] OR Indian*[tiab])) |

Search terms in bold text were removed from the search updates run in 2023 to reduce the large number of search results captured. Socioeconomic status, a topic identified as an established risk factor for cardiovascular disease, had related search terms removed from the disproportionately affected populations search terms for the 2023 search updates.

Table S7. PubMed Literature Search Terms for Targeted Environmental Exposures and Psychosocial Stressors

| Topic | Search Parameters |
| --- | --- |
| Allostatic Load | "allostasis"[MeSH Terms] OR "allostasis"[All Fields] OR ("allostatic"[tiab] AND "load"[tiab]) OR "allostatic load"[All Fields] OR "allostatic loads"[All Fields] |
| Discrimination | "Social Discrimination"[Mesh] OR "Social Segregation"[Mesh] OR ((race[tiab] OR racial*[tiab] OR ethnic[tiab] OR social[tiab] OR perceived[tiab]) AND (discriminat*[tiab] OR bias[tiab] OR segregation[tiab] OR inequity[tiab] OR inequities[tiab] OR inequality[tiab] OR inequalities[tiab])) OR prejudice[tiab] OR racism[tiab] OR racist[tiab] OR racial-isolation[tiab] OR racial-composition[tiab] |
| Heat and Cold | "hot temperature"[mesh] OR "cold temperature"[mesh] OR “extreme heat”[tiab] OR “extreme cold”[tiab] OR “extreme temperature”[tiab] OR “heat extremes”[tiab] OR “cold extremes”[tiab] OR “temperature extremes”[tiab] OR “heat exposure”[tiab] OR “cold exposure”[tiab] OR “heat stress”[tiab] OR “cold stress”[tiab] OR “temperature stress”[tiab] OR “hot weather”[tiab] OR “cold weather”[tiab] OR “heat waves”[tiab] OR “heatwave”[tiab] OR ((“climate change”[tiab] OR “global warming”[tiab]) AND (temperature OR Heat OR Cold)) |
| Noise Pollution | (("Environmental Pollution"[Mesh] AND "Noise"[Mesh]) OR "Noise/adverse effects"[Mesh] OR "noise annoyance"[Tiab] OR "noise pollution"[Tiab] OR "urban noise"[Tiab] OR "city noise"[Tiab] OR "urban sound"[Tiab] OR "environmental noise"[Tiab] OR "community noise"[Tiab] OR "traffic noise"[Tiab] OR "wind turbine noise"[Tiab] OR " wind farm noise"[Tiab] OR " wind turbine sound"[Tiab] OR "wind farm sound"[Tiab] OR " aircraft noise"[Tiab] OR " airport noise"[Tiab] OR " railway noise"[Tiab] OR "road traffic noise"[Tiab] OR "transportation noise"[Tiab] OR " train noise"[Tiab] OR " leisure noise"[Tiab] OR " leisure-time noise"[Tiab] OR "neighbourhood noise"[Tiab] OR "neighborhood noise"[Tiab] OR "household noise"[Tiab] OR "low frequency noise"[Tiab] OR "classroom noise"[Tiab] OR "school noise"[Tiab] OR "high-volume music "[Tiab] OR "high-volume noise"[Tiab] OR "noise from personal electronic devices"[Tiab] OR "noise from mp3 players"[Tiab] OR "noise from childrens toys"[Tiab] OR "hospital noise"[Tiab] OR "combined noise exposure"[Tiab] OR "noise nuisance"[Tiab] OR "noise exposure"[Tiab] OR "truck noise"[Tiab] OR "motor vehicle noise"[Tiab] OR "noise load"[Tiab] OR "entertainment noise"[Tiab] OR "noise from mobile phones"[Tiab] OR "noise from personal audio devices"[Tiab] OR "noise from personal music players"[Tiab] OR "combined exposure to noise and vibration"[Tiab] OR "combined exposure to noise and air pollution"[Tiab]) |
| Shift Work | (work-schedule*[tiab] OR Alternative-shift*[tiab] OR duty-shift*[tiab] OR Midnight-shift*[tiab] OR night-call[tiab] OR night-shift*[tiab] OR nightshift*[tiab] OR night-work*[tiab] OR nightwork*[tiab] OR rotating-schedule*[tiab] OR rotating-shift*[tiab] OR shift-work*[tiab] OR shiftwork*[tiab] OR split-shift*[tiab] OR swing-shift*[tiab] OR third-shift*[tiab]) OR ((“personnel staffing and scheduling”[mh] OR “work schedule tolerance”[mh]) AND (shift* OR schedul*[tiab] OR hours[tiab] OR night[tiab] OR evening[tiab] OR duty-hour*[tiab] OR dutyperiod*[tiab] OR night-float*[tiab] OR overtime[tiab] OR on-call[tiab] OR 12-hour[tiab] OR twelve-hour[tiab] OR "long working hours"[tiab] OR "working long hours"[tiab] OR sleep[tiab] OR fatigue[tiab])) |
| Sleep Quality | "Sleep Hygiene"[Mesh] OR "Sleep Quality"[Mesh] OR "Sleep Quality"[tiab] OR "Sleep Habits"[tiab] OR "Sleep Disturbances"[tiab] OR "Sleep Behavior"[tiab] OR "Sleep Duration"[tiab] OR "Sleep latency"[tiab] |
| Wildfire Smoke | "wildfire smoke"[tiab] OR "Forest fire smoke"[tiab] OR "wildland fire smoke"[tiab] OR ((Wildfire[tiab] OR "Forest fire"[tiab] OR "wildland fire"[tiab] OR wildfire[mesh]) AND ("Smoke"[tiab] OR Smoke[mesh])) |

Literature Screening

Table S8. Inclusion and Exclusion Criteria for Title and Abstract Screening

| Topic | Additional Inclusion Criteria | Additional Exclusion Criteria |
| --- | --- | --- |
| Study Type | Reviews: systematic reviews, meta-analyses of epidemiology studies, agency assessments, and other secondary literature (including non-English language papers)  Primary articles: peer-reviewed primary epidemiology studies (including non-English language papers) | Meeting abstracts, *in vitro* studies, animal studies,*^a^* human but not epidemiology studies (such as intervention studies), and descriptive epidemiology studies (e.g., case reports, case series) |
| Environmental Exposure | General environmental exposures, pollution (as well as specific pollutants), endocrine disruptors, e-waste, chemicals, metals, pesticides, solvents, phthalates, per- and polyfluoroalkyl substances, flame retardants, household products/cosmetics, allergens, noise, and/or diet | Smoking as the main environmental exposure (not a modifier, etc.) |
| Psychosocial Stressor | Psychosocial stressors like discrimination, socioeconomic status, social factors, stress, neighborhood, and/or allostatic load | No additional exclusion criteria |
| Cardiovascular Disease Outcome | Measured cardiovascular disease-related outcomes like heart disease, atherosclerosis, heart attack, stroke, heart failure, arrhythmia, mortality related to cardiovascular disease, and/or heart valve issues in subjects  Cardiovascular disease biomarkers in human epidemiological studies, disability-adjusted life years, and years of life lost | Cardiovascular disease treatment studies |

*^a^*For studies on allostatic load, animal populations were also included.

Table S9. Inclusion and Exclusion Criteria for Full-text Screening

| Topic | Additional Inclusion Criteria | Additional Exclusion Criteria |
| --- | --- | --- |
| Study Type | Reviews: systematic reviews, meta-analyses of epidemiology studies, agency assessments, and other secondary literature (including non-English language papers)  Primary articles: peer-reviewed primary epidemiology studies (including non-English language papers) | Meeting abstracts, *in vitro* studies, animal studies, *^a^* human but not epidemiology studies (such as intervention studies), and descriptive epidemiology studies (e.g., case reports, case series) |
| Environmental Exposure | General environmental exposures, pollution (as well as specific pollutants), endocrine disruptors, e-waste, chemicals, metals, pesticides, solvents, phthalates, per- and polyfluoroalkyl substances, flame retardants, household products/cosmetics, allergens, noise, and/or diet | Smoking as the main environmental exposure (not a modifier, etc.); weather events (e.g., tsunami or Fukoshima nuclear reactor meltdown); exercise, obesity, high body mass index, illegal drug use, and vaccinations |
| Psychosocial Stressor | Psychosocial stressors like discrimination, socioeconomic status, social factors, stress, neighborhood, and/or allostatic load | Depression, military service, posttraumatic stress disorder, religion, spiritual coping, recent medical diagnoses (e.g., HIV AIDS), living at a high altitude, low life satisfaction, hopelessness, sadness, serving as a caregiver |
| Cardiovascular Disease Outcome | Measured cardiovascular disease-related outcomes like heart disease, atherosclerosis, heart attack, stroke, heart failure, arrhythmia, mortality related to cardiovascular disease, and/or heart valve issues in subjects  Cardiovascular disease biomarkers in human epidemiological studies, disability-adjusted life years, and years of life lost | Cardiovascular disease treatment studies, post-cardiovascular disease outcomes (e.g., post-stroke outcomes, stroke rehabilitation), rheumatic heart disease, diabetes, chronic obstructive pulmonary disease; serum measurements like cholesterol, triglycerides, LDL, HDL, which are not specific enough to cardiovascular disease outcomes |

*^a^*For studies on allostatic load, animal populations were also included.

Table S10. Tagging Criteria for Full-text Screening

| Topic | Tagging Criteria |
| --- | --- |
| Study Type | Primary article: peer-reviewed primary human epidemiology studies, including the following study designs: case-control, cohort (including nested case-control studies), cross-sectional, experimental study (except studies that look at CVD treatments or interventions), pooled analysis, risk assessment (e.g., health impact assessment), other study design (e.g., time-series study, ecologic study, crossover study, panel study), and unclear study design  Review: human epidemiology systematic review, meta-analysis, agency assessment, articles that comment on other relevant studies (e.g., discuss a potential issue with a study’s analysis or model) or provides supporting information to help understand a relevant study or other secondary literature that evaluates evidence derived from primary literature sources  Primary animal studies: peer-reviewed animal studies examining allostatic load |
| Study Geography | United States: study occurred in the United States  Other high-income countries: study occurred in a high-income country, as defined by the World Bank [1]  Low- and middle-income countries: study occurred in a low- or middle-income country, as defined by the World Bank [1]  Review (multi-country) or unclear: review that contained data from multiple countries or it is unclear where the study occurred |
| Environmental Exposure | Air pollution**:** indoor air pollution, particulate matter, gaseous pollutants, traffic-related air pollution, wildfire smoke, and other air pollution (e.g., general air pollution not specified further, pollutant standards index)  Allergens  Consumer exposure**:** endocrine disruptors, flame retardants, household products/cosmetics, phthalates, and other consumer exposure (e.g., acrylamide)  Diet**:** alcohol, deficiency, healthy diet, overnutrition, soy intake, and other diet (e.g., dietary nitrates, fatty acid intake, studies on metals in diet that do not look at a deficiency or excess)  Environmental pollutants: electronic waste, per- and polyfluoroalkyl substances, persistent organic pollutants, pesticides, polychlorinated biphenyls, polycyclic aromatic hydrocarbons, solvents, and other environmental pollutants  Metals**:** arsenic, cadmium, copper, lead, mercury, selenium, zinc, and other metals  Occupational exposure  Physical agents**:** heat/cold, noise pollution, radiation, climate-related (e.g., increased hurricanes due to climate, humidity, and other climate-related exposures that do not fit under heat/cold), and other physical agents (e.g., seasonality, light pollution, electromagnetic fields, geomagnetic disturbances, light at night, circadian disruption, daylight savings)  Shift work  Sleep quality  Smoking: environmental tobacco smoke and primary tobacco smoke  Other environmental exposure: environmental exposures that do not fit into the other categories above |
| Psychosocial Stressor | Allostatic load  Discrimination**:** racism, segregation, immigration-related stressors (e.g., fear of deportation, English as a second language, English proficiency), and other discrimination (e.g., everyday discrimination, lifetime discrimination, perceived discrimination)  Education level  Food insecurity: living in a food desert and grocery store density  Geography**:** urban vs. rural location, neighborhood (e.g., neighborhood disorder, neighborhood stress, neighborhood violence, living in slums), and other geography (e.g., regionality, City Human Development Index)  Green space: residential greenness, lack of green space, sky view, percent vegetation cover, and normalized difference vegetation index  SES: individual SES, neighborhood deprivation, neighborhood SES, poverty, health insurance status, housing tenure, housing insecurity, white collar vs. blue collar job, social mobility, Gini coefficient, poverty-income ratio, Index of Multiple Deprivation, and Carstairs index  Work-related stress  Other psychosocial stressor: psychosocial stressors that do not fit the categories above including stress, maternal stress, perceived stress scale, political/election-related stress, health literacy, unemployment, residential mobility, pain interference, social deprivation, childhood adversity index, abuse, parental aggression, incarceration/prison, transportation vulnerability, social cohesion, and non-SES specific vulnerability index |
| CVD Outcome | Organ/subclinical: atherosclerosis, atherosclerosis-related effects, other arterial effects, carotid intimal thickness, arterial calcification, arterial stiffness, changes in ejection function, atrial ejection function, atrial volume, early diastolic transmittal inflow, left atrial volume index, mitral annular velocities, pulmonary artery pressure, ventricular dysfunction, ventricular ejection fraction, ventricular end-diastolic volume, ventricular end-systolic volume, ventricular volume, heart rate variability, electrocardiogram endpoints, heart rate recovery, heart rate variability and changes in heart rate, other heart rate, hypertension/blood pressure measurements, vasoconstriction, myocardial remodeling, left ventricular mass index, ventricular hypertrophy, ventricular mass, sphericity index, vascular/endothelial dysfunction, and flow-mediated dilation  Disease**:** aneurysm, arrythmia, atrial fibrillation, ventricular electrical abnormalities, cardiomyopathy/heart muscle disease, cerebrovascular disease, congenital/developmental heart disease, coronary artery disease, coronary heart disease, acute coronary syndrome, peripheral artery disease, valvular disease, venous thromboembolism, deep vein thrombosis, pulmonary embolism, and other embolism (venous or arterial)  Clinical syndrome**:** angina, cardiac arrest, heart failure, myocardial dysfunction, myocardial infarction/heart attack, and stroke  Pregnancy hypertension**:** preeclampsia, eclampsia, and other pregnancy hypertension  General CVD outcomes**:** CVD-related mortality, CVD-related hospital admissions, CVD incidence, and other general CVD outcomes  Biomarker/allostatic load: allostatic load, general CVD biomarker, inflammation biomarker, oxidative stress biomarker, and other biomarkers  Oxidative stress or inflammation biomarker only: studies in which the only health outcome was oxidative stress or an inflammation biomarker  CVD impacts/CVD + other disease: CVD + other disease and CVD impacts, including disability-adjusted life years and years of life lost  Overall CVD risk |
| Women-specific Studies | Studies comprising only women or, in mixed-sex studies, containing relevant results stratified or separated by sex |
| DAPs*^a^* | Asian: people with origins or ancestry in East Asia, Southeast Asia, or the Indian subcontinent [2]  Black  Hispanic  Indigenous: Native Americans, Inuit, Native Hawaiians, and Aboriginals  Low socioeconomic status  Rural  Sexual minority: gay, lesbian, bisexual, and transgender  Other DAP: Arab Americans, Middle Eastern populations, unspecified ethnic minorities, and studies that looked at general “non-White,” “Brown,” or “of color” populations but did not specify further |

Abbreviations: CVD, cardiovascular disease; SES, socioeconomic status; DAPs, disproportionately affected populations.

*^a^*For studies outside of the United States, DAPs were tagged according to which populations are considered DAPs in those countries.

Supplemental Analysis

Table S11. Summary of Epidemiological Studies Examining the Relationship Between Discrimination and CVD Outcomes

| Type of CVD Outcome | CVD Endpoint  (# of Studies*^a^*) | Study Designs  (# of Studies) | Findings*^b^* | Notes |
| --- | --- | --- | --- | --- |
| Biomarkers | Allostatic load (27 publications, 21 studies) | Longitudinal (4): Brody et al., 2014; Fuller-Rowell et al., 2012; Upchurch et al., 2015; Vadiveloo et al., 2017 [3-6]  Cross-sectional (23 publications, 18 studies): Allen et al., 2019a; Allen et al., 2019b; Cave et al., 2020; Chen at al., 2024; Copeland et al., 2021; Cuevas et al., 2004; Cuevas et al., 2019; Cuevas et al., 2021; Currie et al., 2020; Daniels et al., 2023; de Castro et al., 2010; Doan et al., 2024; Graham et al., 2024; Juster et al., 2024; Lawrence et al., 2022; Obaoye et al., 2023; Ong et al., 2017; Rosemberg et al., 2019; Rosemberg et al., 2022; Seeman et al., 2014; Thomas et al, 2019; Tomfohr et al., 2016; Van Dyke et al, 2020 [7-29] | All studies: 90% positive for direct, indirect, or effect modification; 2 studies (4 publications) unclear  Discrimination (15 studies, 17 publications): >90 % positive (not including subgroup of overlapping population, Rosemberg et al., 2022), one unclear  Segregation: One positive study  Racial discrimination (5 studies, 9 publications): 80% positive, 1 unclear (one inverse analysis and 3 positive analyses from the same study) | Discrimination types: discrimination (including weight and sexual discrimination) and racial discrimination  Exposures: sleep  Effect modification: coping, SES, sleep, institutional belonging  Longitudinal studies: All positive  Findings:   - Positive findings were found across several racial and ethnic groups and in 4 longitudinal studies; most studies in DAP - Johnson et al. [30] looked at components of allostatic load and found complex patterns with discrimination and allostatic load, coping and gender*^c^*   Overlapping populations:   - Sample of Indigenous adults: [9, 11] - Cross-sectional analyses of adults from the Boston Puerto Rican Health Study: [10, 18] - Sample of 30-50 year-old African-American women living in San Francisco: [7, 8, 15, 21] - Sample of hotel housekeepers: [13, 19] (subset of Mexican American immigrant housekeepers) |
| Biomarkers | All biomarkers (22) | Longitudinal (5): Fuller et al., 2019; Sladek et al., 2021; Slopen et al., 2019; Vadiveloo et al., 2017; Zahodne et al., 2019 [6, 31-34]  Cross-sectional (14): Brown et al., 2020; Caceres et al., 2021; Cedillo et al., 2020; Cook et al., 2022; Drolet et al., 2020; Johnson et al., 2022; Lawrence et al., 2022; Merritt et al., 2024;Parra et al., 2020; Priest et al., 2020; Saban et al., 2018; Skinner et al., 2011; Stokes et al., 2020; Wood et al., 2019 [23, 30, 35-46]  Experimental/randomized: exposure to discrimination (3): Arriola et al., 2021; Huebner et al., 2021; Park et al., 2018 [47-49] | All studies: 83% positive for direct, indirect, or effect modification; 2 unclear  Racial discrimination (10): 80% positive for direct, indirect or effect modification; 1 unclear, 1 null  Discrimination (13): 83% positive for direct, indirect or effect modification, 1 unclear, 1 null | Biomarkers included:   - Cortisol (6) [34-36, 44, 47, 48] - C-reactive protein (9) [31-33, 37-39, 43-46] - Other inflammation (8) [6, 23, 30, 40-43, 49] Discrimination types: discrimination, (including sexual orientation), and racial discrimination   Exposures: sleep, alcohol, smoking but did not look at interaction with PSS, air pollution  Effect modifiers: air pollution, other PSS including SES  Findings: Associations were positive across several racial and ethnic groups and in four longitudinal and two experimental studies |
| Biomarkers/ Subclinical | CVD risk/score or ideal CVD*^d^* (23 publications; 21 studies) | Cross-sectional (18 publications, 17 studies): Baxter et al., 2021; Berkowitz et al., 2022; Cameron et al., 2025; Chen et al., 2021; Chilunga et al., 2019; Cohn et al., 2017; Hines et al., 2023; Hussain et al. 2021; Lawrence et al., 2022; Lee et al., 2021; Mereish et al., 2020; Ko et al., 2021; Martos-Méndez et al., 2020; Mujahid et al., 2021; Needham et al., 2025; Shah et al., 2024; Turkson-Ocran et al., 2020; Wassink et al., 2017 [23, 50-66]  Longitudinal (5 publications, 4 populations): Allgood et al.,2024; Bey et al., 2019; Bey et al., 2020; Lamar et al., 2023; Vargas et al., 2022 [67-71] | All studies: 71% positive, 3 unclear  Segregation (3 studies): 2 positive, 1 with mixed results  Racial discrimination (6 studies, 7 publications): all positive  Discrimination (13 studies, 15 publications): 62% positive. 2 unclear | Discrimination types: discrimination, racial discrimination, and segregation  Racial discrimination surrogates: Redlining [56], skin color [57], state laws [65]  Exposures: none  Effect modifiers: race, gender, SES, and other psychosocial stressors  Findings:   - Findings for longitudinal analyses were mainly null (25% positive) - Three publications from Coronary Artery Risk Development in Young Adults (CARDIA) Study; Bey [69] provided a deeper analysis of Bey [68] (null) and found that the setting of racial or gender discrimination may impact CVD health (both counted as one study).Vargas did not find an association with discrimination in a subset of the population that reported cardiometabolic symptoms (counted as a separate study).   Overlapping populations:   - Young adults from the CARDIA Study: [68, 69] - Adults from the MIDUS Study: [23, 50] |
| Subclinical | Atherosclerosis related (9) | Longitudinal (2): Reddy et al., 2022; Udo et al., 2017 [72, 73]  Cross-sectional (7): Ashe et al., 2024; Beatty Moody et al., 2020; Bromfield et al., 2020; Camelo et al., 2022; Cook et al., 2024; Lewis et al., 2019; Okhomina, 2018 [74-80] | All studies: 80% positive for direct effects or indirect effects, 1 unclear  Racial discrimination and segregation (4): 100% positive for direct effects  Discrimination (6): 67% for direct effects or indirect effects, 1 unclear | Discrimination types: discrimination (including weight and sex), racial discrimination, and segregation  Exposures: none  Findings:   - Positive findings found in Brown/Black people or Black women, and one study of all adults - Nonsignificant findings in discrimination study (weight and gender) counted as positive [73] - Positive findings in 2 cohort studies - Timing of segregation may be important |
| Subclinical | Cardiac related (7); vascular dysfunction (1);  changes in ejection function (1) | Cross-sectional (5): Hagen et al., 2021; Hicken et al., 2016; Hill et al., 2017; Martin et al., 2023; van Nieuwenhuizen et al., 2021; [81-85]  Experimental/ randomized: exposure to discrimination (4): Cavanagh et al., 2022; Hermosura et al., 2018; Huebner et al., 2021; Williams et al., 2019 [48, 86-88] | Cardiac related: 86% positive for direct effects or effect modification   - Discrimination (2): 50% positive for direct effects - Racial discrimination (5): all positive for direct effects or effect modification; 80% for direct effects   Vascular function: null [85]  Changes in ejection function: null for effect modification with air pollution [84] | Discrimination types: racial discrimination, segregation, and discrimination (including sexual orientation)  Exposures: air pollution  Effect modification: air pollution, other stressors, coping  Findings: negative cardiac-related study looks at a different measurement than the positive studies (heart rate variability) and is for discrimination |
| Subclinical | Hypertension, blood pressure, and related measurements (76 publications, 70 studies) | Longitudinal (25 publications, 22 studies): Barber et al., 2018; Beatty Moody et al., 2018; Caceres et al., 2023; Chirinos et al., 2024; Clausing et al., 2021; Coates et al., 2024; D'Agostino et al., 2018a; D'Agostino et al., 2018b; D'Agostino et al., 2021; Forde et al., 2020; Forde et al., 2021; Fox et al., 2019; Friedman et al., 2022; Gao et al., 2022; Gaston et al., 2023; Gero et al., 2022; Kim et al., 2022; Mendes et al., 2020; Moody et al, 2019; Reges et al, 2023; Sims et al., 2022; Sladek et al., 2021; Udo et al., 2017; Vadiveloo et al., 2017; Vargas et al., 2022 [6, 34, 67, 73, 89-109]  Cross-sectional (40 publications, 37 studies): Adil et al., 2022; Ashe et al., 2024; Barajas et al., 2019; Basile Ibrahim et al., 2021; Cedillo et al., 2020; Chan et al., 2024; Dawson et al., 2021; De et al., 2020; Gabriel et al., 2020; Hines et al., 2018; Hsiao et al., 2022; Johnson et al., 2022; Lawrence et al., 2023; LeBrón et al., 2020; Lee et al., 2019; Loose et al., 2017; Hill et al., 2018; Mendes et al., 2018; Mereish et al., 2020; Michaels et al., 2019a; Michaels et al., 2019b; Mujahid et al., 2021; Nguyen et al., 2022; Pössel et al., 2023; Priest et al., 2020; Reeves et al., 2024; Resnicow et al., 2021; Scott et al., 2020; Shin et al., 2017; Siddiqi et al., 2017; Taylor et al., 2017; Teteh et al., 2020; Thayer et al., 2017; Thomas Tobin et al., 2022; Thurber et al., 2021; Turkson-Ocran et al., 2020; Usher et al., 2018; Wassink et al., 2017; Wright et al., 2020; Zawadzki et al., 2023 [30, 43, 44, 56, 57, 61, 62, 110-143]  Experimental, randomized or other (11): Arriola et al., 2021; Hermosura et al., 2018; Huang et al., 2020; Huang et al., 2022; Huebner et al,, 2021; Jones et al., 2024; Mohottige et al., 2023; Motairek et al., 2022; Siegel et al., 2023a; Siegel et al., 2023b; Stanhope et al., 2024 [48, 49, 88, 144-151] | All studies (70): 77% positive (any discrimination) for direct effects or effect modification; 2 unclear  Racial discrimination (30 studies, 33 publications): 83% positive for direct effects or effect modification findings for 1 study unclear  Segregation (9 studies, 10 publications): 67% positive for direct effects or effect modification  Discrimination (36 studies, 37 publications): ~72% positive for direct effects or effect modification, one unclear | Discrimination types: discrimination (including institutional, interpersonal, gender, weight, and sexual orientation) racial discrimination, and segregation  Racial discrimination surrogates: redlining [56, 145], Jones et al. 2024, Chan et al. 2024 negative tweets [148], living in an area with hate crimes [102], policies or practices [110, 146], skin color [57]  Intervention: segregation studies [96, 97]  Exposures: sleep, smoking, alcohol, diet  Effect modifiers: coping, unfairness, genetics,  Findings:   - Cohort studies: ~70% positive, one unclear - Stronger association in Black people (most studied) - Less consistency of findings across studies by the type of discrimination (e.g., every day, lifetime)   Overlapping populations:   - Participants in the ELSA-Brazil study: Cross-sectional analysis [117], cohort [95] - Participants in the African American Women’s Heart and Health Study: [118, 133, 141] - Adults from the Study of Women’s Health Across the Nation: [90, 104] - Children attending the Fit2Play afterschool program in Miami, FL: [96, 97] - Sister Study: Coates et al., 2024; Gaston et al., 2023 [108, 109] |
| Diseases/Event: All | CVD incidence, mortality, or any CVD (25) | Longitudinal (including case-cohort) (11): Ajibewa et al., 2025; Al-Kindi et al., 2023; Bhavsar et al., 2021; Cannavale et al., 2024; Cummings et al., 2021; Deng et al., 2024; Freedman et al., 2022; Hussein et al., 2018; Kim et al., 2022; Lawrence et al., 2023, Murphy et al., 2024 [103, 139, 152-160]  Ecological or other designs (6): Huang et al., 2020; Islami et al., 2021; Kramer et al., 2017; Kyalwazi et al., 2022; Yitshak-Sade et al., 2020; Zestcott et al., 2021 [148, 161-165]  Cross-sectional (8): Avery et al., 2024; Cuevas et al., 2021; Forrester et al., 2021; Merritt et al., 2024; Muchomba et al., 2024; Poteat et al., 2021; Reddy et al., 2023; Thurber et al., 2021 [46, 130, 166-171] | All studies:76% positive, 1 unclear  Racial discrimination (10): 90% positive, 1 unclear, 1 null  Segregation (7): 86% for direct or effect modification, 1 unclear  Discrimination (8): 63% positive. Two studies, one positive for CVD mortality among Black people (Lawrence et al. 2023), and one null for CVD incident among all subjects (incidence and mortality, Hussein et al. 2018) used data from the same cohort. | Discrimination types: discrimination, racial discrimination (including holding prejudice attitudes), and segregation  Racial discrimination surrogates: living in neighborhoods with excessive police force [156, 171], negative twitter tweets [148], redlining [153], history of slavery [163]  Exposures and effect modifiers: 1 study evaluated the interaction of PM2.5 and segregation  Findings: cohort studies were 64% positive (including effect modification) |
| Diseases | Coronary artery disease (CAD) (9)*^f^* | Longitudinal (4): Dunlay et al., 2017; Jackson et al., 2019; Norland et al., 2024; Sheehy et al., 2024 [172-175]  Ecological or related studies (5): Huang et al., 2020; Huang et al., 2022; Jones et al., 2024; Motairek et al., 2022; West et al., 2024 [144, 145, 148, 149, 176] | All studies: 67% positive for direct effects  Racial discrimination (4): 100% positive  Discrimination (3): 67% positive  Segregation (2): 1 null, 1 unclear | Discrimination types: racial discrimination, segregation, and discrimination (including age, and health discrimination)  Racial discrimination surrogates: negative tweets [148], redlining [145, 149]  Exposures: none  Cohort studies: 75% positive |
| Diseases | Hypertensive diseases of pregnancy (13) | Longitudinal (5): Caplan et al., 2021; Everett et al., 2024; Frances et al., 2024; Grobman et al., 2018; Janevic et al., 2025 [177-181]  Cross-sectional (7): Chan et al., 2024; Christian et al., 2021; Lee et al., 2025; Mayne et al., 2018; Muchomba et al., 2024; Stanhope et al., 2023; Walker et al., 2024 [142, 171, 182-186]  Other design (1): Stanhope et al., 2024 [151] | All studies: 62% positive  Racial discrimination (8): 50% positive  Discrimination (4): 50% positive  Segregation (2): All positive | Discrimination types: racial, discrimination, and segregation  Racial discrimination surrogates: practices or polices [184], historical slavery [151], redlining [142], excessive police force  Exposures: none  Longitudinal studies: 80% positive |
| Diseases | Other specific CVD (4) | Longitudinal (2): Al-Kindi et al., 2023; Murosko et al., 2020 [153, 187]  Cross-sectional (2): Adebiyi et al., 2023; Poteat et al., 2021 [166, 188] | Venous thromboembolism: 1 positive study [166]  Children’s or developmental heart disease: 2 positive studies [187, 188]  Atrial fibrillation and peripheral artery disease: 1 null study [153] | Discrimination types: racial discrimination and other discrimination  Racial discrimination surrogate: redlining [153]  Exposures: none  Findings: positive results identified in the cohort study for developmental heart disease but not for the adult diseases (in both cross-sectional and prospective analysis) |
| Clinical Events | Heart failure (5) | Longitudinal (3): Al-Kindi et al., 2023; Dunlay et al., 2017; Fields et al., 2024 [153, 172, 189]  Cross-sectional (1): Mentias et al., 2023 [190]  Ecological (1): Splan et al., 2021 [191] | All studies: 80% positive  Racial discrimination/segregation (4): 100% positive  Discrimination (1): null | Discrimination types: racial and other discrimination  Racial discrimination surrogates: redlining [153, 189]  Exposures: none  Longitudinal studies: 2 positive, 1 null  Findings: stronger findings among Black people and mixed finding in cohort studies |
| Clinical Events | Myocardial Infarction (4) | Longitudinal (3): Al-Kindi et al., 2023; Sheehy et al., 2024; Udo et al., 2017 [73, 153, 175]  Other study design (1): Huang et al., 2020 [148] | All studies: 100% positive  Racial discrimination (4): 100% positive  Discrimination (1): positive | Discrimination types: racial, weight and sex  Racial discrimination surrogates: negative tweets and redlining [148, 153]  Exposures: none  Findings: results were positive for weight-based and racial discrimination but not sex-based discrimination in the cohort reported by Udo et al. [73] |
| Diseases/ Events: Clinical Events | Stroke (16) | Longitudinal (4): Al-Kindi et al., 2023; Dunlay et al., 2017; Jackson et al., 2019; Sheehy et al., 2024 [153, 172, 173, 192]  Cross-sectional (2): Forrester et al., 2021; Trifan et al., 2023 [169, 193]  Ecological or other study design (10): Gompers et al., 2025; Huang et al., 2020; Huang et al., 2022; Jadow et al., 2023; Jones et al., 2025; Motairek et al., 2022; Siegel et al., 2023a; Siegel et al., 2023b; Span et al., 2021; Wing et al., 2022 [144-149, 191, 194-196] | All studies: 75% positive; 1 unclear  Racial discrimination (12): 83% positive, 1 unclear, 1 null  Discrimination (2): 50% positive  Segregation (3): 67% | Discrimination types: racial discrimination, discrimination (including age discrimination), and segregation  Racial surrogates: redlining [145, 149, 153, 194, 195], negative tweets [148], and combined proxies [146]  Exposures: none  Findings:   - Cohort studies were 50% positive - Discrimination studies: age discrimination was positive, and everyday discrimination was null (2 separate studies) |

Abbreviations: CVD, cardiovascular disease; DAP, disproportionately affected population; PSS, psychosocial stressor; SES, socioeconomic status.

*^a^* ‘Publication’ refers to a peer-reviewed article submitted to a scientific journal, and ‘study’ refers to an analysis of a given exposure and outcome in a population examined by one or more publications. Publications reported on the same exposure, outcome, and population are considered as one study, e.g., more detailed analyses of the exposure. For the purpose of this review, we considered analyses of very specific subsets of the publication as separate studies unless there was likely to be a large overlap.

^b^Findings were considered positive for either direct (e.g., discrimination associated with CVD) or indirect effects (e.g., association observed for effect modification or pathway analyses). Positive percentages were counted for studies and not publication

*^c^*This study was not included in results totals because it looked at individual components of allostatic load

*^d^*CVD risk/score or ideal CVD is based on a composite of biomarkers.

*^e^*Findings from the two publications were considered positive based on an in-depth analysis.

*^f^*Wing et al. (2022) reported on CAD but is not included in total because no CAD analyses were conducted.

References

1. The World Bank. World Bank country and lending groups. 2021. <https://datahelpdesk.worldbank.org/knowledgebase/articles/906519-world-bank-country-and-lending-groups>. Accessed 18 Feb 2021.

2. U.S. Census Bureau. About the topic of race. 2021. U.S. Census Bureau. About the topic of race. 2021. <https://www.census.gov/topics/population/race/about.html#:~:text=Asian%20%E2%80%93%20A%20person%20having%20origins,Islands%2C%20Thailand%2C%20and%20Vietnam>. Accessed 18 Feb 2021.

3. Brody GH, Lei MK, Chae DH, Yu T, Kogan SM, Beach SRH. Perceived discrimination among African American adolescents and allostatic load: a longitudinal analysis with buffering effects. Child Dev. 2014;85(3):989-1002.

4. Fuller-Rowell TE, Evans GW, Ong AD. Poverty and health: The mediating role of perceived discrimination. Psychol Sci. 2012;23(7):734-9.

5. Upchurch DM, Stein J, Greendale GA, Chyu L, Tseng CH, Huang MH, Lewis TT, Kravitz HM, Seeman T. A longitudinal investigation of race, socioeconomic status, and psychosocial mediators of allostatic load in midlife women: Findings from the study of women's health across the nation. Psychosom Med. 2015;77(4):402-12.

6. Vadiveloo M, Mattei J. Perceived weight discrimination and 10-year risk of allostatic load among US adults. Ann Behav Med. 2017;51(1):94-104.

7. Allen AM, Wang Y, Chae DH, Price MM, Powell W, Steed TC, Rose Black A, Dhabhar FS, Marquez-Magaña L, Woods-Giscombe CL. Racial discrimination, the superwoman schema, and allostatic load: exploring an integrative stress-coping model among African American women. Ann N Y Acad Sci. 2019;1457(1):104-27.

8. Allen AM, Thomas MD, Michaels EK, Reeves AN, Okoye U, Price MM, Hasson RE, Syme SL, Chae DH. Racial discrimination, educational attainment, and biological dysregulation among midlife African American women. Psychoneuroendocrinology. 2019;99:225-35.

9. Copeland JL, Currie CL, Chief Moon-Riley K. Physical activity buffers the adverse impacts of racial discrimination on allostatic load among Indigenous adults. Ann Behav Med. 2021;55(6):520-9.

10. Cuevas AG, Wang K, Williams DR, Mattei J, Tucker KL, Falcon LM. The association between perceived discrimination and allostatic load in the Boston Puerto Rican Health Study. Psychosom Med. 2019;81(7):659-67.

11. Currie CL, Motz T, Copeland JL. The impact of racially motivated housing discrimination on allostatic load among Indigenous university students. J Urban Health. 2020;97(3):365-76.

12. Ong AD, Williams DR, Nwizu U, Gruenewald TL. Everyday unfair treatment and multisystem biological dysregulation in African American adults. Cultur Divers Ethnic Minor Psychol. 2017;23(1):27-35.

13. Rosemberg MS, Li Y, McConnell DS, McCullagh MC, Seng JS. Stressors, allostatic load, and health outcomes among women hotel housekeepers: A pilot study. J Occup Environ Hyg. 2019;16(3):206-17.

14. Seeman M, Stein Merkin S, Karlamangla A, Koretz B, Seeman T. Social status and biological dysregulation: the "status syndrome" and allostatic load. Soc Sci Med. 2014;118:143-51.

15. Thomas MD, Michaels EK, Reeves AN, Okoye U, Price MM, Hasson RE, Chae DH, Allen AM. Differential associations between everyday versus institution-specific racial discrimination, self-reported health, and allostatic load among black women: implications for clinical assessment and epidemiologic studies. Ann Epidemiol. 2019;35:20-8.e3.

16. Tomfohr LM, Pung MA, Dimsdale JE. Mediators of the relationship between race and allostatic load in African and White Americans. Health Psychol. 2016;35(4):322-32.

17. Van Dyke ME, Baumhofer NK, Slopen N, Mujahid MS, Clark CR, Williams DR, Lewis TT. Pervasive discrimination and allostatic load in African American and white adults. Psychosom Med. 2020;82(3):316-23.

18. Cuevas AG, Abuelezam NN, Chan SWC, Carvalho K, Flores C, Wang K, Mattei J, Tucker KL, Falcon LM. Skin tone, discrimination, and allostatic load in middle-aged and older Puerto Ricans. Psychosom Med. 2021;83(7):805-12.

19. Rosemberg MS, Li Y, Polick C. Immigration-related stressors and health outcomes among low-wage immigrant hotel workers: A pilot study. Public Health Nurs. 2022;39(5):1123-7.

20. Cave L, Cooper MN, Zubrick SR, Shepherd CCJ. Racial discrimination and allostatic load among First Nations Australians: A nationally representative cross-sectional study. BMC Public Health. 2020;20(1):1881.

21. Daniels KP, D Thomas M, Chae DH, Allen AM. Black mothers' concern for their children as a measure of vicarious racism-related vigilance and allostatic load. J Health Soc Behav. 2023;64(4):520-36.

22. de Castro AB, Voss JG, Ruppin A, Dominguez CF, Seixas NS. Stressors among Latino day laborers. A pilot study examining allostatic load. Aaohn j. 2010;58(5):185-96.

23. Lawrence JA, Kawachi I, White K, Bassett MT, Williams DR. Associations between multiple indicators of discrimination and allostatic load among middle-aged adults. Soc Sci Med. 2022;298(114866):114866.

24. Obaoye JO, Dawson AZ, Thorgerson A, Ikonte CO, Williams JS, Egede LE. Understanding the relationship between perceived discrimination, allostatic load, and all-cause mortality in US older adults: A mediation analysis. J Am Geriatr Soc. 2023;71(5):1515-25.

25. Chen JC, Handley D, Elsaid MI, Plascak JJ, Andersen BL, Carson WE, Pawlik TM, Carlos RC, Obeng-Gyasi S. The Implications of Racialized Economic Segregation and Allostatic Load on Mortality in Patients with Breast Cancer.  Ann Surg Oncol. 2024;31(1):365-375.

26. Cuevas AG, McSorley AM, Lyngdoh A, Kaba-Diakité F, Harris A, Rhodes-Bratton B, Rouhani S. Education, Income, Wealth, and Discrimination in Black-White Allostatic Load Disparities.  Am J Prev Med. 2024;67(1):97-104.

27. Doan S, Davis AS, Lazarus M, Poudel A, Tran P, Clark N, Fuller-Rowell TE. Belonging Exacerbates the Relations Between Racial Climate Stress and Physiological Dysregulation.  J Racial Ethn Health Disparities. 2024;11(5):2786-2795.

28. Graham C. Accumulating burden: Exposure to interpersonal discrimination based on multiple attributes and allostatic load. 2024(2352-8273 (Print)).

29. Juster RP, Rutherford C Fau - Keyes K, Keyes K Fau - Hatzenbuehler ML, Hatzenbuehler ML. Associations Between Structural Stigma and Allostatic Load Among Sexual Minorities: Results From a Population-Based Study. Psychosom Med. 2024;86(3):157-168.

30. Johnson AJ, McCloyn K, Sims M. Discrimination, high-effort coping, and cardiovascular risk profiles in the Jackson Heart Study: A latent profile analysis. J Racial Ethn Health Disparities. 2022;9(4):1464-73.

31. Fuller CH, Appleton AA, Bulsara PJ, O'Neill MS, Chang HH, Sarnat JA, Falcón LM, Tucker KL, Brugge D. Sex differences in the interaction of short-term particulate matter exposure and psychosocial stressors on C-reactive protein in a Puerto Rican cohort. SSM Popul Health. 2019;9:100500.

32. Slopen N, Strizich G, Hua S, Gallo LC, Chae DH, Priest N, Gurka MJ, Bangdiwala SI, Bravin JI, Chambers EC et al. Maternal experiences of ethnic discrimination and child cardiometabolic outcomes in the Study of Latino Youth. Ann Epidemiol. 2019;34:52-7.

33. Zahodne LB, Kraal AZ, Zaheed A, Farris P, Sol K. Longitudinal effects of race, ethnicity, and psychosocial disadvantage on systemic inflammation. SSM Popul Health. 2019;7:100391.

34. Sladek MR, Castro SA, Doane LD. Ethnic-Racial discrimination experiences predict Latinx adolescents' physiological stress processes across college transition. Psychoneuroendocrinology. 2021;128:105212.

35. Parra LA, Hastings PD. Challenges to identity integration indirectly link experiences of heterosexist and racist discrimination to lower waking salivary cortisol in sexually diverse Latinx emerging adults. Front Psychol. 2020;11:228.

36. Skinner ML, Shirtcliff EA, Haggerty KP, Coe CL, Catalano RF. Allostasis model facilitates understanding race differences in the diurnal cortisol rhythm. Dev Psychopathol. 2011;23(4):1167-86.

37. Cook SH, Slopen N, Scarimbolo L, Mirin N, Wood EP, Rosendale N, Chunara R, Burke CW, Halkitis PN. Discrimination is associated with C-reactive protein among young sexual minority men. Journal of behavioral medicine. 2022.

38. Drolet CE, Lucas T. Perceived racism, affectivity, and C-reactive protein in healthy African Americans: Do religiosity and racial identity provide complementary protection? J Behav Med. 2020;43(6):932-42.

39. Stokes JE. Social integration, daily discrimination, and biological markers of health in mid- and later life: Does self-esteem play an intermediary role? Innov Aging. 2020;4(4):igaa026.

40. Saban KL, Mathews HL, Bryant FB, Tell D, Joyce C, DeVon HA, Witek Janusek L. Perceived discrimination is associated with the inflammatory response to acute laboratory stress in women at risk for cardiovascular disease. Brain Behav Immun. 2018;73:625-32.

41. Brown KM, Diez-Roux AV, Smith JA, Needham BL, Mukherjee B, Ware EB, Liu Y, Cole SW, Seeman TE, Kardia SLR. Social regulation of inflammation related gene expression in the multi-ethnic study of atherosclerosis. Psychoneuroendocrinology. 2020;117:104654.

42. Caceres BA, Barcelona V, Vo D, Suero-Tejeda N, Jackman K, Taylor J, Corwin E. Investigating the associations of everyday discrimination and inflammation in Latina women: A pilot study. Biological research for nursing. 2021;23(3):311-7.

43. Priest N, Truong M, Chong S, Paradies Y, King TL, Kavanagh A, Olds T, Craig JM, Burgner D. Experiences of racial discrimination and cardiometabolic risk among Australian children. Brain Behav Immun. 2020;87:660-5.

44. Cedillo YE, Lomax RO, Fernandez JR, Moellering DR. Physiological significance of discrimination on stress markers, obesity, and LDL oxidation among a European American and African American cohort of females. Int J Behav Med. 2020;27(2):213-24.

45. Wood EP, Cook SH. Father support is protective against the negative effects of perceived discrimination on CRP among sexual minorities but not heterosexuals. Psychoneuroendocrinology. 2019;110(104368):104368.

46. Merritt C, Muscatell K. Discrimination and Cardiovascular Health in Black Americans: Exploring Inflammation as a Mechanism and Perceived Control as a Protective Factor. Psychosom Med. 2024;86(3):181-191.

47. Park J, Flores AJ, Aschbacher K, Mendes WB. When anger expression might be beneficial for African Americans: The moderating role of chronic discrimination. Cultur Divers Ethnic Minor Psychol. 2018;24(3):303-18.

48. Huebner DM, McGarrity LA, Perry NS, Spivey LA, Smith TW. Cardiovascular and cortisol responses to experimentally-induced minority stress. Health psychology : official journal of the Division of Health Psychology, American Psychological Association. 2021;40(5):316-25.

49. Arriola KJ, Lewis TT, Pearce B, Cobb J, Weldon B, Valentin MIZ, Lea J, Vaccarino V. A randomized trial of race-related stress among African Americans with chronic kidney disease. Psychoneuroendocrinology. 2021;131(105339):105339.

50. Lee C, Park S, Boylan JM. Cardiovascular health at the intersection of race and gender: Identifying life-course processes to reduce health disparities. J Gerontol B Psychol Sci Soc Sci. 2021;76(6):1127-39.

51. Chilunga FP, Boateng D, Henneman P, Beune E, Requena-Méndez A, Meeks K, Smeeth L, Addo J, Bahendeka S, Danquah I et al. Perceived discrimination and stressful life events are associated with cardiovascular risk score in migrant and non-migrant populations: The RODAM study. Int J Cardiol. 2019;286:169-74.

52. Chen S, Mallory AB. The effect of racial discrimination on mental and physical health: A propensity score weighting approach. Social science & medicine (1982). 2021;285:114308.

53. Baxter SLK, Chung R, Frerichs L, Thorpe RJ, Jr., Skinner AC, Weinberger M. Racial residential segregation and race differences in ideal cardiovascular health among young men. International journal of environmental research and public health. 2021;18(15).

54. Cohn T, Miller A, Fogg L, Braun LT, Coke L. Impact of individual and neighborhood factors on cardiovascular risk in white Hispanic and Non-Hispanic women and men. Res Nurs Health. 2017;40(2):120-31.

55. Martos-Méndez MJ, García-Cid A, Gómez-Jacinto L, Hombrados-Mendieta I. Perceived discrimination, psychological distress and cardiovascular risk in migrants in Spain. International journal of environmental research and public health. 2020;17(12).

56. Mujahid MS, Gao X, Tabb LP, Morris C, Lewis TT. Historical redlining and cardiovascular health: The Multi-Ethnic Study of Atherosclerosis. Proceedings of the National Academy of Sciences of the United States of America. 2021;118(51).

57. Wassink J, Perreira KM, Harris KM. Beyond race/ethnicity: Skin color and cardiometabolic health among Blacks and Hispanics in the United States. J Immigr Minor Health. 2017;19(5):1018-26.

58. Berkowitz J, Khetpal V, Echouffo-Tcheugui JB, Bambs CE, Aiyer A, Kip KE, Reis SE, Erqou S. Associations between cumulative social risk, psychosocial risk, and ideal cardiovascular health: Insights from the HeartSCORE study. Am J Prev Cardiol. 2022;11(100367):100367.

59. Hines AL, Albert MA, Blair JP, Crews DC, Cooper LA, Long DL, Carson AP. Neighborhood factors, individual stressors, and cardiovascular health among Black and White adults in the US: The Reasons for Geographic and Racial Differences in Stroke (REGARDS) study. JAMA Netw Open. 2023;6(9):e2336207.

60. Ko Y-A, Shen J, Kim JH, Topel M, Mujahid M, Taylor H, Quyyumi A, Sims M, Vaccarino V, Baltrus P et al. Identifying neighbourhood and individual resilience profiles for cardiovascular health: a cross-sectional study of blacks living in the Atlanta metropolitan area. BMJ Open. 2021;11(7):e041435.

61. Mereish EH, Goldstein CM. Minority stress and cardiovascular disease risk among sexual minorities: Mediating effects of sense of mastery. Int J Behav Med. 2020;27(6):726-36.

62. Turkson-Ocran R-AN, Szanton SL, Cooper LA, Golden SH, Ahima RS, Perrin N, Commodore-Mensah Y. Discrimination is associated with elevated cardiovascular disease risk among African immigrants in the African Immigrant Health Study. Ethn Dis. 2020;30(4):651-60.

63. Hussain M, Howell JL, Peek MK, Stowe RP, Zawadzki MJ. Psychosocial stressors predict lower cardiovascular disease risk among Mexican-American adults living in a high-risk community: Findings from the Texas City Stress and Health Study. PloS one. 2021;16(10):e0257940.

64. Cameron N, Huang X, Petito L, Ning H, Shah N, Yee L, Perak A, Haas D, Mercer BM, Parry S et al. Determinants of Racial and Ethnic Differences in Maternal Cardiovascular Health in Early Pregnancy. Circ Cardiovasc Qual Outcomes. 2025;18(3):e011217.

65. Needham B, Dokshina D, Ali T, Allgood K, Douglas M, Dulin A, Fleischer N, Johnson A, Ro A, Agénor M. Exposure to structural racism-related state laws is associated with worse cardiovascular health among US adults, BRFSS 2011 and 2013.  Am J Epidemiol. 2025;194(1):142-151.

66. Shah NS, Huang X, Petito LC, Bancks MP, Kanaya AM, Talegawkar S, Farhan S, Carnethon MR, Lloyd-Jones DM, Allen NB et al. Social and psychosocial determinants of racial and ethnic differences in cardiovascular health: The MASALA and MESA studies. Am J Prev Cardiol. 2024;17:100636.

67. Vargas EA, Chirinos DA, Wong M, Carnethon MR, Carroll AJ, Kiefe CI, Carson AP, Kershaw KN. Psychosocial profiles and longitudinal achievement of optimal cardiovascular risk factor levels: the Coronary Artery Risk Development in Young Adults (CARDIA) study. Journal of behavioral medicine. 2022;45(2):172-85.

68. Bey GS, Jesdale B, Forrester S, Person SD, Kiefe C. Intersectional effects of racial and gender discrimination on cardiovascular health vary among black and white women and men in the CARDIA study. SSM Popul Health. 2019;8:100446.

69. Bey GS, Person SD, Kiefe C. Gendered race and setting matter: Sources of complexity in the relationships between reported interpersonal discrimination and cardiovascular health in the CARDIA Study. Journal of racial and ethnic health disparities. 2020;7(4):687-97.

70. Lamar M, Estrella ML, Capuano AW, Leurgans S, Fleischman DA, Barnes LL, Lange-Maia BS, Bennett DA, Marquez DX. A longitudinal study of acculturation in context and cardiovascular health and their effects on cognition among older Latino adults. J Am Heart Assoc. 2023;12(6):e027620.

71. Allgood K, Fleischer N, Assari S, Morenoff J, Needham B. School Segregation During Adolescence is Associated with Higher 30-Year Cardiovascular Risk of Black but not White Young Adults. J Racial Ethn Health Disparities. 2024;12(5):3324-3338.

72. Reddy NM, Mayne SL, Pool LR, Gordon-Larsen P, Carr JJ, Terry JG, Kershaw KN. Exposure to neighborhood-level racial residential segregation in young adulthood to midlife and incident subclinical atherosclerosis in Black adults: The Coronary Artery Risk Development in Young Adults Study. Circulation Cardiovascular quality and outcomes. 2022;15(2):e007986.

73. Udo T, Grilo CM. Cardiovascular disease and perceived weight, racial, and gender discrimination in U.S. adults. J Psychosom Res. 2017;100:83-8.

74. Beatty Moody DL, Leibel DK, Pantesco EJ, Wendell CR, Waldstein SR, Evans MK, Zonderman AB. Interactive relations across dimensions of interpersonal-level discrimination and depressive symptoms to carotid intimal-medial thickening in African Americans. Psychosom Med. 2020;82(2):234-46.

75. Bromfield SG, Sullivan S, Saelee R, Elon L, Lima B, Young A, Uphoff I, Li L, Quyyumi A, Bremner JD et al. Race and gender differences in the association between experiences of everyday discrimination and arterial stiffness among patients with coronary heart disease. Annals of behavioral medicine : a publication of the Society of Behavioral Medicine. 2020;54(10):761-70.

76. Camelo LV, Machado AV, Chor D, Griep RH, Mill JG, Brant LCC, Barreto SM. Racial discrimination is associated with greater arterial stiffness and carotid intima-media thickness: The ELSA-Brasil study. Annals of epidemiology. 2022.

77. Lewis TT, Lampert R, Charles D, Katz S. Expectations of racism and carotid intima-media thickness in African American women. Psychosom Med. 2019;81(8):759-68.

78. Okhomina VI, Glover L, Taylor H, Sims M. Dimensions of and responses to perceived discrimination and subclinical disease among African-Americans in the Jackson Heart Study. J Racial Ethn Health Disparities. 2018;5(5):1084-92.

79. Ashe J, MacIver PH, Sun S, Taylor AD, Evans MK, Zonderman AB, Waldstein SR. Discrimination, religious affiliation, and arterial stiffness in African American women and men. Health Psychol. 2024;43(12):853-862.

80. Cook S, Wood E, Stein J, McClelland R. Discrimination, Smoking, and Cardiovascular Disease Risk: A Moderated Mediation Analysis With MESA. J Am Heart Assoc. 2024;13(5):e032659.

81. Hagen JM, Sutterland AL, Collard D, de Jonge CDE, van Zuiden M, Zantvoord JB, Tan HL, van Valkengoed IGM, van den Born BJH, Zwinderman AH et al. Ethnic discrimination and depressed mood: The role of autonomic regulation. J Psychiatr Res. 2021;144:110-7.

82. van Nieuwenhuizen BP, Sekercan A, Tan HL, Blom MT, Lok A, van den Born BH, Kunst AE, van Valkengoed IGM. Is the association between education and sympathovagal balance mediated by chronic stressors? International journal of behavioral medicine. 2021.

83. Hill LK, Hoggard LS, Richmond AS, Gray DL, Williams DP, Thayer JF. Examining the association between perceived discrimination and heart rate variability in African Americans. Cultur Divers Ethnic Minor Psychol. 2017;23(1):5-14.

84. Hicken MT, Adar SD, Hajat A, Kershaw KN, Do DP, Barr RG, Kaufman JD, Diez Roux AV. Air pollution, cardiovascular outcomes, and social disadvantage: The Multi-Ethnic Study of Atherosclerosis. Epidemiology. 2016;27(1):42-50.

85. Martin ZT, Al-Daas IO, Cardenas N, Kolade JO, Merlau ER, Vu JK, Brown KK, Brothers RM. Peripheral and cerebral vascular reactivity in Black and white women: Examining the impact of psychosocial stress exposure versus internalization and coping. medRxiv. 2023.

86. Williams DP, Joseph N, Hill LK, Sollers JJ, 3rd, Vasey MW, Way BM, Koenig J, Thayer JF. Stereotype threat, trait perseveration, and vagal activity: evidence for mechanisms underpinning health disparities in Black Americans. Ethn Health. 2019;24(8):909-26.

87. Cavanagh L, Obasi EM. Chronic stress, autonomic dysregulation and prospective drug use among African American emerging adults. Cultur Divers Ethnic Minor Psychol. 2022;28(1):91-102.

88. Hermosura AH, Haynes SN, Kaholokula JK. A preliminary study of the relationship between perceived racism and cardiovascular reactivity and recovery in Native Hawaiians. J Racial Ethn Health Disparities. 2018;5(5):1142-54.

89. Barber S, Diez Roux AV, Cardoso L, Santos S, Toste V, James S, Barreto S, Schmidt M, Giatti L, Chor D. At the intersection of place, race, and health in Brazil: Residential segregation and cardio-metabolic risk factors in the Brazilian Longitudinal Study of Adult Health (ELSA-Brasil). Soc Sci Med. 2018;199:67-76.

90. Beatty Moody DL, Chang Y, Brown C, Bromberger JT, Matthews KA. Everyday discrimination and metabolic syndrome incidence in a racially/ethnically diverse sample: Study of women's health across the nation. Psychosom Med. 2018;80(1):114-21.

91. Forde AT, Sims M, Muntner P, Lewis T, Onwuka A, Moore K, Diez Roux AV. Discrimination and hypertension risk among African Americans in the Jackson Heart Study. Hypertension (Dallas, Tex : 1979). 2020;76(3):715-23.

92. Forde AT, Lewis TT, Kershaw KN, Bellamy SL, Diez Roux AV. Perceived discrimination and hypertension risk among participants in the multi-ethnic study of atherosclerosis. Journal of the American Heart Association. 2021;10(5):e019541.

93. Gao X, Kershaw KN, Barber S, Schreiner PJ, Do DP, Diez Roux AV, Mujahid MS. Associations between residential segregation and incident hypertension: The Multi-Ethnic Study of Atherosclerosis. Journal of the American Heart Association. 2022;11(3):e023084.

94. Fox RS, Carnethon MR, Gallo LC, Wiley JF, Isasi CR, Daviglus ML, Cai J, Davis SM, Giachello AL, Gonzalez P et al. Perceived discrimination and cardiometabolic risk among US Hispanics/Latinos in the HCHS/SOL Sociocultural Ancillary Study. Int J Behav Med. 2019;26(4):331-42.

95. Mendes PM, Nobre AA, Griep RH, Juvanhol LL, Barreto SM, Fonseca MJM, Chor D. Association between race/color and incidence of hypertension in the ELSA-Brasil population: investigating the mediation of racial discrimination and socioeconomic position. Ethnicity & health. 2020:1-11.

96. D'Agostino EM, Patel HH, Ahmed Z, Hansen E, Sunil Mathew M, Nardi MI, Messiah SE. Impact of change in neighborhood racial/ethnic segregation on cardiovascular health in minority youth attending a park-based afterschool program. Soc Sci Med. 2018;205:116-29.

97. D'Agostino EM, Patel HH, Ahmed Z, Hansen E, Mathew MS, Nardi MI, Messiah SE. Natural experiment examining the longitudinal association between change in residential segregation and youth cardiovascular health across race/ethnicity and gender in the USA. J Epidemiol Community Health. 2018;72(7):595-604.

98. D'Agostino EM, Patel HH, Hansen E, Mathew MS, Messiah SE. Longitudinal effects of transportation vulnerability on the association between racial/ethnic segregation and youth cardiovascular health. Journal of racial and ethnic health disparities. 2021;8(3):618-29.

99. Caceres BA, Sharma Y, Levine A, Wall MM, Hughes TL. Investigating the associations of sexual minority stressors and incident hypertension in a community sample of sexual minority adults. Ann Behav Med. 2023;57(12):1004-13.

100. Clausing ES, Non AL. Epigenetics as a mechanism of developmental embodiment of stress, resilience, and cardiometabolic risk across generations of Latinx immigrant families. Front Psychiatry. 2021;12:696827.

101. Friedman MR, Liu Q, Meanley S, Haberlen SA, Brown AL, Turan B, Turan JM, Brennan-Ing M, Stosor V, Mimiaga MJ et al. Biopsychosocial health outcomes and experienced intersectional stigma in a mixed HIV serostatus longitudinal cohort of aging sexual minority men, United States, 2008‒2019. Am J Public Health. 2022;112(S4):S452-S62.

102. Gero K, Noubary F, Kawachi I, Baum CF, Wallace RB, Briesacher BA, Kim D. Associations of state-level and county-level hate crimes with individual-level cardiovascular risk factors in a prospective cohort study of middle-aged Americans: the National Longitudinal Survey of Youths 1979. BMJ Open. 2022;12(1):e054360.

103. Kim MH, Schwartz GL, White JS, Glymour MM, Reardon SF, Kershaw KN, Gomez SL, Collin DF, Inamdar PP, Wang G et al. School racial segregation and long-term cardiovascular health among Black adults in the US: A quasi-experimental study. PLoS Med. 2022;19(6):e1004031.

104. Moody DLB, Chang Y-F, Pantesco EJ, Darden TM, Lewis TT, Brown C, Bromberger JT, Matthews KA. Everyday discrimination prospectively predicts blood pressure across 10 years in racially/ethnically diverse midlife women: Study of Women's Health Across the Nation. Ann Behav Med. 2019;53(7):608-20.

105. Reges O, Krefman AE, Hardy ST, Yano Y, Muntner P, Pool LR, Gordon-Larsen P, Wang Y, Lloyd-Jones DM, Allen NB. Race- and sex-specific factors associated with age-related slopes in systolic blood pressure: Findings from the CARDIA study. Hypertension. 2023;80(9):1890-9.

106. Sims KD, Smit E, Batty GD, Hystad PW, Odden MC. Intersectional discrimination and change in blood pressure control among older adults: The Health and Retirement Study. J Gerontol A Biol Sci Med Sci. 2022;77(2):375-82.

107. Chirinos DA, Vargas EA, Kershaw KN, Wong M, Everson-Rose SA. Psychosocial profiles and blood pressure control: results from the multi-ethnic study of atherosclerosis (MESA).  J Behav Med. 2024;47(6):1067-1079.

108. Coates M, Arah OA, Matthews TA, Sandler DP, Jackson CL, Li J. Multiple forms of perceived job discrimination and hypertension risk among employed women: Findings from the Sister Study. Am J Ind Med. 2024;67(9):844-856.

109. Gaston SA, Forde AT, Green M, Sandler DP, Jackson CL. Racial and Ethnic Discrimination and Hypertension by Educational Attainment Among a Cohort of US Women. JAMA Netw Open. 2023;6(11):e2344707.

110. Basile Ibrahim B, Barcelona V, Condon EM, Crusto CA, Taylor JY. The association between neighborhood social vulnerability and cardiovascular health risk among Black/African American women in the InterGEN Study. Nursing research. 2021;70(5S Suppl 1):S3-s12.

111. Dawson AZ, Walker RJ, Gregory C, Egede LE. Quantifying direct effects of social determinants of health on systolic blood pressure in United States adult immigrants. Journal of behavioral medicine. 2021;44(3):345-54.

112. Gabriel AC, Bell CN, Bowie JV, Hines AL, LaVeist TA, Thorpe RJ, Jr. The association between perceived racial discrimination and hypertension in a low-income, racially integrated urban community. Family & community health. 2020;43(2):93-9.

113. Hines AL, Pollack CE, LaVeist TA, Thorpe RJ, Jr. Race, vigilant coping strategy, and hypertension in an integrated community. Am J Hypertens. 2018;31(2):197-204.

114. LeBrón AMW, Schulz AJ, Mentz G, Reyes AG, Gamboa C, Israel BA, Viruell-Fuentes EA, House JS. Impact of change over time in self-reported discrimination on blood pressure: implications for inequities in cardiovascular risk for a multi-racial urban community. Ethn Health. 2020;25(3):323-41.

115. Loose F, Tiboulet M, Maisonneuve C, Taillandier-Schmitt A, Dambrun M. Blood pressure and psychological distress among North Africans in France: The role of perceived personal/group discrimination and gender. Am J Hum Biol. 2017;29(5).

116. Hill LK, Sherwood A, McNeilly M, Anderson NB, Blumenthal JA, Hinderliter AL. Impact of racial discrimination and hostility on adrenergic receptor responsiveness in African American adults. Psychosom Med. 2018;80(2):208-15.

117. Mendes PM, Nobre AA, Griep RH, Guimarães JMN, Juvanhol LL, Barreto SM, Pereira A, Chor D. Association between perceived racial discrimination and hypertension: findings from the ELSA-Brasil study. Cad Saude Publica. 2018;34(2):e00050317.

118. Michaels E, Thomas M, Reeves A, Price M, Hasson R, Chae D, Allen A. Coding the everyday discrimination scale: Implications for exposure assessment and associations with hypertension and depression among a cross section of mid-life African American women. J Epidemiol Community Health. 2019;73(6):577-84.

119. Resnicow K, Patel M, Green M, Smith A, Bacon E, Goodell S, Kilby D, Tariq M, Alhawli A, Syed N et al. The association of unfairness with mental and physical health in a multiethnic sample of adults: Cross-sectional study. JMIR Public Health Surveill. 2021;7(5):e26622.

120. Scott J, Silva S, Simmons LA. Social adversity, sleep characteristics, and elevated blood pressure among young adult Black females. Health equity. 2020;4(1):421-9.

121. Shin CN, Soltero E, Mama SK, Sunseri C, Lee RE. Association of discrimination and stress with cardiometabolic risk factors in ethnic minority women. Clin Nurs Res. 2017;26(6):694-712.

122. Siddiqi A, Shahidi FV, Ramraj C, Williams DR. Associations between race, discrimination and risk for chronic disease in a population-based sample from Canada. Soc Sci Med. 2017;194:135-41.

123. Taylor JY, Sun YV, Barcelona de Mendoza V, Ifatunji M, Rafferty J, Fox ER, Musani SK, Sims M, Jackson JS. The combined effects of genetic risk and perceived discrimination on blood pressure among African Americans in the Jackson Heart Study. Medicine (Baltimore). 2017;96(43):e8369.

124. Thayer ZM, Blair IV, Buchwald DS, Manson SM. Racial discrimination associated with higher diastolic blood pressure in a sample of American Indian adults. Am J Phys Anthropol. 2017;163(1):122-8.

125. Usher T, Gaskin DJ, Bower K, Rohde C, Thorpe RJ, Jr. Residential segregation and hypertension prevalence in Black and white older adults. J Appl Gerontol. 2018;37(2):177-202.

126. Wright ML, Lim S, Sales A, Rajagopal S, Nzegwu D, Crusto CA, Taylor JY. The influence of discrimination and coping style on blood pressure among Black/African American women in the InterGEN Study. Health equity. 2020;4(1):272-9.

127. Barajas CB, Jones SCT, Milam AJ, Thorpe RJ, Jr., Gaskin DJ, LaVeist TA, Furr-Holden CDM. Coping, discrimination, and physical health conditions among predominantly poor, urban African Americans: Implications for community-level health services. J Community Health. 2019;44(5):954-62.

128. Adil O, Kuk JL, Ardern CI. Associations between weight discrimination and metabolic health: A cross sectional analysis of middle aged adults. Obes Res Clin Pract. 2022;16(2):151-7.

129. Gaston SA, Jackson WB, 2nd, Williams DR, Jackson CL. Sleep and cardiometabolic health by government-assisted rental housing status among Black and White men and women in the United States. Sleep Health. 2018;4(5):420-8.

130. Thurber KA, Colonna E, Jones R, Gee GC, Priest N, Cohen R, Williams DR, Thandrayen J, Calma T, Lovett R et al. Prevalence of everyday discrimination and relation with wellbeing among Aboriginal and Torres Strait Islander adults in Australia. Int J Environ Res Public Health. 2021;18(12):6577.

131. De PK. Beyond race: Impacts of non-racial perceived discrimination on health access and outcomes in New York City. PloS one. 2020;15(9):e0239482.

132. Hsiao CJ, Dumeny L, Bress AP, Johnson DA, Shimbo D, Cavallari LH, Mulligan CJ. Identification of a SGCD × discrimination interaction effect on systolic blood pressure in African American adults in the Jackson Heart Study. Am J Hypertens. 2022;35(11):938-47.

133. Michaels EK, Reeves AN, Thomas MD, Price MM, Hasson RE, Chae DH, Allen AM. Everyday racial discrimination and hypertension among midlife African American women: Disentangling the role of active coping dispositions versus active coping behaviors. Int J Environ Res Public Health. 2019;16(23).

134. Nguyen AW, Miller D, Bubu OM, Taylor HO, Cobb R, Trammell AR, Mitchell UA. Discrimination and hypertension among older African Americans and Caribbean Blacks: The moderating effects of John Henryism. J Gerontol B Psychol Sci Soc Sci. 2022;77(11):2049-59.

135. Pössel P, Dondanville AA. Role of dysfunctional attitudes in the association between discrimination and adolescents' mental and physical health. Psychol Rep. 2023:332941231186801.

136. Teteh DK, Lee JW, Montgomery SB, Wilson CM. Working together with God: Religious coping, perceived discrimination, and hypertension. J Relig Health. 2020;59(1):40-58.

137. Thomas Tobin CS, Gutiérrez Á, Norris KC, Thorpe RJ. Discrimination, racial identity, and hypertension among Black Americans across young, middle, and older adulthood. J Gerontol B Psychol Sci Soc Sci. 2022;77(11):1990-2005.

138. Zawadzki MJ, Graham-Engeland JE, Robles PL, Hussain M, Fair EV, Tobin JN, Cassells A, Brondolo E. Acute experiences of negative interpersonal interactions: Examining the dynamics of negative mood and ambulatory blood pressure responses among Black and Hispanic urban adults. Ann Behav Med. 2023;57(8):630-9.

139. Lawrence WR, Jones GS, Johnson JA, Ferrell KP, Johnson JN, Shiels MS, Diez Roux AV, Forde AT. Discrimination experiences and all-cause and cardiovascular mortality: Multi-ethnic study of atherosclerosis. Circ Cardiovasc Qual Outcomes. 2023;16(4):e009697.

140. Lee AK, Corneille MA, Brandon DT. Masculinity ideology and racism as indicators of obesity risk among Black men. J Natl Med Assoc. 2019;111(6):642-7.

141. Reeves A, Michaels EK, Thomas MD, Okoye U, Price MM, Hasson RE, Chae DH, Allen AM. All stressors are not equal: The salience of racial discrimination and appraisal for blood pressure in African American women. Psychosom Med. 2024;86(1):20-9.

142. Chan M, Parikh S, Willcocks E, Lytel-Sternberg J, Castro E, Tabb LP, Schwartz J, James-Todd T. Associations between Historical Redlining and the Risk of Pregnancy Complications and Adverse Birth Outcomes in Massachusetts, 1995-2015. J Womens Health (Larchmt). 2024;33(10):1308-1317.

143. Ashe J, Bentley-Edwards K, Skipper A, Cuevas A, Vieytes CM, Bah K, Evans MK, Zonderman AB, Waldstein SR. Racial Discrimination, Religious Coping, and Cardiovascular Disease Risk Among African American Women and Men. J Racial Ethn Health Disparities. 2024;12(5):3069-3085.

144. Huang H. Moderating effects of racial segregation on the associations of cardiovascular outcomes with walkability in Chicago metropolitan area. Int J Environ Res Public Health. 2022;19(21):14252.

145. Motairek I, Lee EK, Janus S, Farkouh M, Freedman D, Wright J, Nasir K, Rajagopalan S, Al-Kindi S. Historical neighborhood redlining and contemporary cardiometabolic risk. J Am Coll Cardiol. 2022;80(2):171-5.

146. Siegel M, Rieders M, Rieders H, Moumneh J, Asfour J, Oh J, Oh S. Structural racism and racial health disparities at the state level: A latent variable approach. J Natl Med Assoc. 2023;115(4):338-52.

147. Siegel M, Wiklund E. The relationship between state-level structural racism and disparities between the non-hispanic black and non-hispanic white populations in multiple health outcomes. J Natl Med Assoc. 2023;115(2):207-22.

148. Huang D, Huang Y, Adams N, Nguyen TT, Nguyen QC. Twitter-characterized sentiment towards racial/ethnic minorities and cardiovascular disease (CVD) outcomes. J Racial Ethn Health Disparities. 2020;7(5):888-900.

149. Jones E, Natale B, Blatt L, Votruba-Drzal E, Miller P, Marsland A, Sadler R. Historical Structural Racism in the Built Environment and Physical Health among Residents of Allegheny County, Pennsylvania. J Urban Health. 2024;101(4):713-729.

150. Mohottige D, Davenport CA, Bhavsar N, Schappe T, Lyn MJ, Maxson P, Johnson F, Planey AM, McElroy LM, Wang V et al. Residential Structural Racism and Prevalence of Chronic Health Conditions. JAMA Netw Open. 2023;6(12):e2348914.

151. Stanhope K, Kramer M, McKinnon I, Carter S, Boulet S. Slavery, homeownership, and contemporary perinatal outcomes in the southeast: a test of mediation and moderation. Am J Epidemiol. 2024;193(12):1684-1692.

152. Cummings DM, Patil SP, Long DL, Guo B, Cherrington A, Safford MM, Judd SE, Howard VJ, Howard G, Carson AP. Does the association between hemoglobin A(1c) and risk of cardiovascular events vary by residential segregation? The REasons for Geographic And Racial Differences in Stroke (REGARDS) Study. Diabetes Care. 2021;44(5):1151-8.

153. Al-Kindi S, Motairek I, Kreatsoulas C, Wright JT, Jr., Dobre M, Rahman M, Rajagopalan S. Historical neighborhood redlining and cardiovascular risk in patients with chronic kidney disease. Circulation. 2023;148(3):280-2.

154. Bhavsar NA, Davenport CA, Yang LZ, Peskoe S, Scialla JJ, Hall RK, Tyson CC, Strigo T, Sims M, Pendergast J et al. Psychosocial determinants of cardiovascular events among black Americans with chronic kidney disease or associated risk factors in the Jackson heart study. BMC nephrology. 2021;22(1):375.

155. Hussein M, Diez Roux AV, Mujahid MS, Hastert TA, Kershaw KN, Bertoni AG, Baylin A. Unequal exposure or unequal vulnerability? Contributions of neighborhood conditions and cardiovascular risk factors to socioeconomic inequality in incident cardiovascular disease in the Multi-Ethnic Study of Atherosclerosis. Am J Epidemiol. 2018;187(7):1424-37.

156. Freedman AA, Papachristos AV, Smart BP, Keenan-Devlin LS, Khan SS, Borders A, Kershaw KN, Miller GE. Complaints about excessive use of police force in women's neighborhoods and subsequent perinatal and cardiovascular health. Sci Adv. 2022;8(3):eabl5417.

157. Ajibewa T, Colangelo L, Chirinos D, Kershaw K, Carnethon M, Allen N. Person-Centered Approach to Understanding Psychosocial Stressor Subgroups and Cardiovascular Disease: New Perspectives From the Multi-Ethnic Study of Atherosclerosis Study. J Am Heart Assoc. 2025;14(2):e038844.

158. Cannavale K, Xu L, Nau C, Armenian S, Bhatia S, Wong FL, Huang P, Cooper R, Chao C. Neighborhood factors associated with late effects among survivors of adolescent and young adult cancer. J Cancer Surviv. 2024;19(6):1806-1820(epub).

159. Deng K, Xu M, Sahinoz M, Cai Q, Shrubsole MJ, Lipworth L, Gupta DK, Dixon DD, Zheng W, Shah R et al. Associations of neighborhood sociodemographic environment with mortality and circulating metabolites among low-income black and white adults living in the southeastern United States.  BMC Med. 2024;22(1):249.

160. Murphy B, Nam Y, McClelland R, Acquah I, Cainzos-Achirica M, Nasir K, Post W, Aldrich M, DeFilippis A. Addition of Social Determinants of Health to Coronary Heart Disease Risk Prediction: The Multi-Ethnic Study of Atherosclerosis. J Am Heart Assoc. 2024;13(14):e033651.

161. Zestcott CA, Ruiz JM, Tietje KR, Stone J. The relationship between racial prejudice and cardiovascular disease mortality risk at the state and county Level. Annals of behavioral medicine : a publication of the Society of Behavioral Medicine. 2021.

162. Islami F, Fedewa SA, Thomson B, Nogueira L, Yabroff KR, Jemal A. Association between disparities in intergenerational economic mobility and cause-specific mortality among Black and White persons in the United States. Cancer epidemiology. 2021;74:101998.

163. Kramer MR, Black NC, Matthews SA, James SA. The legacy of slavery and contemporary declines in heart disease mortality in the U.S. South. SSM Popul Health. 2017;3:609-17.

164. Kyalwazi AN, Loccoh EC, Brewer LC, Ofili EO, Xu J, Song Y, Joynt Maddox KE, Yeh RW, Wadhera RK. Disparities in cardiovascular mortality between Black and White adults in the United States, 1999 to 2019. Circulation. 2022;146(3):211-28.

165. Yitshak-Sade M, Lane KJ, Fabian MP, Kloog I, Hart JE, Davis B, Fong KC, Schwartz JD, Laden F, Zanobetti A. Race or racial segregation? Modification of the PM2.5 and cardiovascular mortality association. PloS one. 2020;15(7):e0236479.

166. Poteat TC, Divsalar S, Streed CG, Jr., Feldman JL, Bockting WO, Meyer IH. Cardiovascular disease in a population-based sample of transgender and cisgender adults. Am J Prev Med. 2021;61(6):804-11.

167. Reddy KP, Eberly LA, Julien HM, Giri J, Fanaroff AC, Groeneveld PW, Khatana SAM, Nathan AS. Association between racial residential segregation and Black-White disparities in cardiovascular disease mortality. Am Heart J. 2023;264:143-52.

168. Cuevas AG, Ho T, Rodgers J, DeNufrio D, Alley L, Allen J, Williams DR. Developmental timing of initial racial discrimination exposure is associated with cardiovascular health conditions in adulthood. Ethnicity & health. 2021;26(7):949-62.

169. Forrester SN, Zmora R, Schreiner PJ, Jacobs DR, Jr., Roger VL, Thorpe RJ, Jr., Kiefe CI. Accelerated aging: A marker for social factors resulting in cardiovascular events? SSM Popul Health. 2021;13(100733):100733.

170. Avery L, Maddox R, Abtan R, Wong O, Rotondi NK, McConkey S, Bourgeois C, McKnight C, Wolfe S, Flicker S et al. Modelling prevalent cardiovascular disease in an urban Indigenous population. Can J Public Health. 2024;115(Suppl 2):288-300.

171. Muchomba FM, Teitler JO, Fox C, Reichman NE. Racialized Police Use of Force and Maternal Health. Am J Prev Med. 2024;68(3):535-544(epub).

172. Dunlay SM, Lippmann SJ, Greiner MA, O'Brien EC, Chamberlain AM, Mentz RJ, Sims M. Perceived discrimination and cardiovascular outcomes in older African Americans: Insights from the Jackson Heart Study. Mayo Clin Proc. 2017;92(5):699-709.

173. Jackson SE, Hackett RA, Steptoe A. Associations between age discrimination and health and wellbeing: cross-sectional and prospective analysis of the English Longitudinal Study of Ageing. Lancet Public Health. 2019;4(4):e200-e8.

174. Norland K, Schaid DJ, Naderian M, Na J, Kullo IJ. Associations of Self-Reported Race, Social Determinants of Health, and Polygenic Risk With Coronary Heart Disease. J Am Coll Cardiol. 2024;84(22):2157-2166.

175. Sheehy S, Brock M, Palmer J, Albert M, Cozier Y, Rosenberg L. Perceived Interpersonal Racism in Relation to Incident Coronary Heart Disease Among Black Women. Circulation. 2024;149(7):521-528.

176. West K, Allen EM, Neiwert R, LaPlante A, Durben AN, Delgado-Palma V. Lasting Legacy: The Enduring Relationship Between Racially Restrictive Housing Covenants and Health and Wellbeing.  J Urban Health. 2024;101(5):1026-1036.

177. Caplan M, Keenan-Devlin LS, Freedman A, Grobman W, Wadhwa PD, Buss C, Miller GE, Borders AEB. Lifetime psychosocial stress exposure associated with hypertensive disorders of pregnancy. Am J Perinatol. 2021;38(13):1412-9.

178. Grobman WA, Parker CB, Willinger M, Wing DA, Silver RM, Wapner RJ, Simhan HN, Parry S, Mercer BM, Haas DM et al. Racial disparities in adverse pregnancy outcomes and psychosocial stress. Obstet Gynecol. 2018;131(2):328-35.

179. Everett BG, Philbin MM, Homan P. Structural heteropatriarchy and maternal cardiovascular morbidities. Soc Sci Med. 2024;351(Suppl 1):116434.

180. Francis B Fau - Pearl M, Pearl M Fau - Colen C, Colen C Fau - Shoben A, Shoben A Fau - Sealy-Jefferson S, Sealy-Jefferson S. Racial and Economic Segregation Over the Life Course and Incident Hypertensive Disorders of Pregnancy Among Black Women in California. Am J Epidemiol. 2024;193(2):277-284.

181. Janevic T, Howell F, Burdick M, Nowlin S, Maru S, Boychuk N, Oshewa O, Monterroso M, McCarthy K, Gundersen D et al. Racism and Postpartum Blood Pressure in a Multiethnic Prospective Cohort. Hypertension. 2025;82(2):206-215.

182. Christian LM, Koenig J, Williams DP, Kapuku G, Thayer JF. Impaired vasodilation in pregnant African Americans: Preliminary evidence of potential antecedents and consequences. Psychophysiology. 2021;58(1):e13699.

183. Mayne SL, Yellayi D, Pool LR, Grobman WA, Kershaw KN. Racial residential segregation and hypertensive disorder of pregnancy among women in Chicago: Analysis of electronic health record data. Am J Hypertens. 2018;31(11):1221-7.

184. Stanhope KK, Kapila P, Umerani A, Hossain A, Abu-Salah M, Singisetti V, Carter S, Boulet SL. Political representation and perinatal outcomes to Black, White, and Hispanic people in Georgia: a cross-sectional study. Ann Epidemiol. 2023;87:38-44.e2.

185. Walker SL, Walker RJ, Palatnik A, Dawson AZ, Williams JS, Egede LE. Examining the Relationship between Social Determinants of Health and Adverse Pregnancy Outcomes in Black Women. Am J Perinatol. 2024;41(S 01):e2326-e2335.

186. Lee K, Pellowski JA, Brayboy LM, Thompson KD, Dunsiger S. The Association of Racism and Discrimination in Disparities of Hypertensive Disorders of Pregnancy in the United States: An Analysis of PRAMS Data. Matern Child Health J. 2024;28(5):969-978.

187. Murosko D, Passerella M, Lorch S. Racial segregation and intraventricular hemorrhage in preterm infants. Pediatrics. 2020;145(6):e20191508.

188. Adebiyi E, Pietri-Toro J, Awujoola A, Gwynn L. Association of adverse Childhood Experiences with heart conditions in children: Insight from the 2019-2020 national survey of children's health. Children (Basel). 2023;10(3).

189. Fields N, Tristan Urrutia A, Morris A, Kramer MR, Lewis T, Patel S. Historical Redlining and Heart Failure Outcomes Following Hospitalization in the Southeastern United States. J Am Heart Assoc. 2024;13(8):e032019.

190. Mentias A, Mujahid MS, Sumarsono A, Nelson RK, Madron JM, Powell-Wiley TM, Essien UR, Keshvani N, Girotra S, Morris AA et al. Historical redlining, socioeconomic distress, and risk of heart failure among Medicare beneficiaries. Circulation. 2023;148(3):210-9.

191. Splan ED, Magerman AB, Forbes CE. Associations of regional racial attitudes with chronic illness in the United States. Soc Sci Med. 2021;281(114077):114077.

192. Sheehy S, Aparicio HJ, Palmer JR, Cozier Y, Lioutas VA, Shulman JG, Rosenberg L. Perceived Interpersonal Racism and Incident Stroke Among US Black Women. JAMA Netw Open. 2023;6(11):e2343203.

193. Trifan G, Gallo LC, Lamar M, Garcia-Bedoya O, Perreira KM, Pirzada A, Talavera GA, Smoller SW, Isasi CR, Cai J et al. Association of unfavorable social determinants of health with stroke/transient ischemic attack and vascular risk factors in Hispanic/Latino adults: Results from Hispanic Community Health Study/Study of Latinos. J Stroke. 2023;25(3):361-70.

194. Jadow BM, Hu L, Zou J, Labovitz D, Ibeh C, Ovbiagele B, Esenwa C. Historical redlining, social determinants of health, and stroke prevalence in communities in New York City. JAMA Netw Open. 2023;6(4):e235875.

195. Wing JJ, Lynch EE, Laurent SE, Mitchell B, Richardson J, Meier HCS. Historic redlining in Columbus, Ohio associated with stroke prevalence. J Stroke Cerebrovasc Dis. 2022;31(12):106853.

196. Gompers A, Lewis TT, Kramer MR. Structural racism and racial disparities in stroke mortality in the United States, 2021. Soc Sci Med. 2025;366:117705.
